# Cerebellar Oxidative DNA Damage and Altered DNA Methylation in the BTBR T+tf/J Mouse Model of Autism and Similarities with Human Post Mortem Cerebellum

**DOI:** 10.1371/journal.pone.0113712

**Published:** 2014-11-25

**Authors:** Svitlana Shpyleva, Samuil Ivanovsky, Aline de Conti, Stepan Melnyk, Volodymyr Tryndyak, Frederick A. Beland, S. Jill James, Igor P. Pogribny

**Affiliations:** 1 Department of Pediatrics, University of Arkansas for Medical Sciences, Little Rock, Arkansas, United States of America; 2 Division of Biochemical Toxicology, National Center for Toxicological Research, U.S. Food and Drug Administration, Jefferson, Arkansas, United States of America; 3 Emory University, Atlanta, Georgia, United States of America; Rikagaku Kenkyūsho Brain Science Institute, Japan

## Abstract

The molecular pathogenesis of autism is complex and involves numerous genomic, epigenomic, proteomic, metabolic, and physiological alterations. Elucidating and understanding the molecular processes underlying the pathogenesis of autism is critical for effective clinical management and prevention of this disorder. The goal of this study is to investigate key molecular alterations postulated to play a role in autism and their role in the pathophysiology of autism. In this study we demonstrate that DNA isolated from the cerebellum of BTBR T+tf/J mice, a relevant mouse model of autism, and from human post-mortem cerebellum of individuals with autism, are both characterized by an increased levels of 8-oxo-7-hydrodeoxyguanosine (8-oxodG), 5-methylcytosine (5mC), and 5-hydroxymethylcytosine (5hmC). The increase in 8-oxodG and 5mC content was associated with a markedly reduced expression of the 8-oxoguanine DNA-glycosylase 1 (*Ogg1*) and increased expression of *de novo* DNA methyltransferases 3a and 3b (*Dnmt3a* and *Dnmt3b*). Interestingly, a rise in the level of 5hmC occurred without changes in the expression of ten-eleven translocation expression *1 (Tet1)* and *Tet2* genes, but significantly correlated with the presence of 8-oxodG in DNA. This finding and similar elevation in 8-oxodG in cerebellum of individuals with autism and in the BTBR T+tf/J mouse model warrant future large-scale studies to specifically address the role of *OGG1* alterations in pathogenesis of autism.

## Introduction

Autism is a clinically complex, heterogeneous, and behaviorally-defined neurodevelopmental disorder characterized by impaired social skills, communication, and repetitive behaviors. Substantial effort has been devoted in recent years to uncover the underlying mechanisms of genomic, epigenomic, proteomic, metabolic, and physiological alterations associated with the disease development. Although the role of genetic factors in autism has been extensively studied, the genetic variations are extremely heterogeneous and their phenotypic penetrance is highly variable in different individuals [1]–[3]. In addition to genetic alterations, mounting evidence indicates a key role of other molecular and physiological abnormalities, including immune dysregulation, neuroinflammation, epigenetics, oxidative stress, and mitochondrial dysfunction [4]; however, the underlying molecular pathogenesis of autism remains elusive.

In recent years, the cerebellum has emerged as one of the key brain regions affected in autism [5]. This is evidenced by several well-established observations indicating the essential role of autism in the development of basic social capabilities [6], its involvement in extensive neural networks that govern the social, communication, repetitive/restrictive behaviors [7], and motor and cognitive deficits impaired in autism [8].

Elucidating and understanding the molecular processes underlying the pathogenesis of autism is critical for effective clinical management and prevention of this disorder. Investigation of these mechanisms using human subjects is desirable; however, epidemiological and imaging studies are not able to address causality or the molecular underpinnings of the disease. Animal models that resemble core human autistic symptoms may substantially overcome the limitations of human studies and have the flexibility to provide important additional clues regarding the etiology and molecular pathogenesis of autism. Several mouse models have been developed to simulate autism symptoms in humans. Among all mouse models, the inbred BTBR T+tf/J mice is one of the most relevant and commonly used animal models to study autism because they exhibit an autism-like behavioral phenotype [9]–[11]. In addition to behavioral similarities, multiple studies have shown that BTBR T+tf/J mice share many similarities in neuroanatomical and physiological features found in individuals with autism [12]–[14].

Escalating evidence suggests that oxidative stress and aberrations in the cellular epigenome, especially aberrant DNA methylation, are key molecular features of autistic phenotype [15], [16] that are linked to alterations in glutathione metabolism and folate-dependent trans-methylation and trans-sulfuration pathways [17]–[22]. While alterations causing these pathological signs are of great importance, little is known about the underlying mechanisms responsible for their persistence in the pathophysiology of autism.

Based on these considerations, the goal of this study was to evaluate key molecular alterations postulated to play a role in autism and their role in the pathophysiology of autism using inbred BTBR T+tf/J mice, a strain that exhibits an autism-like behavioral phenotype, including deficits in reciprocal social interactions and social approach, and high levels of repetitive self-grooming behavior [9]–[11] in contrast to C57BL/6J mice, a mouse strain characterized by a high sociability and low grooming behavior [14]. In this study, we demonstrate that DNA isolated from the cerebellum of BTBR T+tf/J mice and from human post-mortem cerebellum of individuals with autism, are both characterized by an increased levels of 8-oxo-7-hydrodeoxyguanosine (8-oxodG), 5-methylcytosine (5mC), and 5-hydroxymethylcytosine (5hmC). The increased 8-oxodG and 5mC content were associated with a markedly reduced expression of the 8-oxoguanine DNA-glycosylase 1 (*Ogg1*) and increased expression of *de novo* DNA methyltransferases 3a and 3b (*Dnmt3a* and *Dnmt3b*). Interestingly, a rise in the level of 5hmC occurred without changes in the expression of ten-eleven translocation expression *1 (Tet1)* and *Tet2* genes, but significantly correlated with the presence of 8-oxodG in DNA.

## Materials and Methods

### Mouse and Human Cerebellar samples

The frozen cerebellum from male and female BTBR T+tf/J and C57BL/6J mice (8 weeks of age, n = 5 per gender/strain) were obtained from the Jackson Laboratory (Bar Harbor, ME, USA). The frozen blocks of post-mortem cerebellum from autism individuals (n = 15) and unaffected control individuals (n = 15) were obtained from the National Institute of Child Health and Development Brain Tissue Bank for Developmental Disorders at the University of Maryland, and from the Autism Tissue Program at the Harvard Brain Tissue Resource Center, Bellmont, MA, USA. All donors had a confirmed diagnosis of autism based on Diagnostic and Statistical Manual of Mental Disorders and Diagnostic Interview Revised. Autism and control groups were matched, as closely as possible, for post-mortem interval, age, gender, race, and cause of death. The demographic data of case-control tissue samples are detailed in James *et al.* [23]. The analysis of postmortem brain specimens is not defined as human research by the United State Department of Health and Health Services (DHHS) and Food and Drug Administration (FDA) regulations.

### Analysis of 8-oxo-7-hydrodeoxyguanosine, 5-methylcytosine, and 5-hydroxymethylcytosine in cerebellar DNA

The levels of 8-oxodG, 5mC, and 5hmC in mouse and human cerebellar DNA were measured by liquid chromatography combined with electrospray tandem mass spectrometry (LC-MS/MS) as described previously [22], [23].

### Determination of mitochondrial DNA damage

The extent of mitochondrial DNA damage was determined by measuring the mitochondrial DNA and genomic DNA (mtDNA/gDNA) ratio in the mouse DNA samples by using the NovaQUANT Mouse Mitochondrial to Nuclear DNA Ratio Kit (EMD Millipore, Billerica, MA, USA) according to the manufacturer's protocol. Briefly, simultaneous analysis of copy number of the beclin 1 (*Becn1*) and nebulin (*Neb*) genes and mitochondrial 12S rRNA gene and mitochondrial DNA fragment trLEU was accomplished by quantitative PCR (qPCR) for each DNA sample, and the mtDNA/nDNA ratio was calculated by using the relative copy number method.

### RNA extraction and gene expression analysis using microarray technology

Total RNA was extracted from a cerebellum tissue using RNeasy Mini kits (Qiagen, Valencia, CA, USA) according to the manufacturer's instructions. Gene expression profiles in the cerebellum of BTBR T+tf/J and C57BL/6J mice were determined utilizing Agilent whole genome 8×60 K mouse microarrays (Agilent Technologies, Santa Clara, CA, USA). Sample labeling and microarray processing were performed as detailed in the “One-Color Microarray-Based Gene Expression Analysis” Version 5.5 (Agilent Technologies) protocol. The hybridized slides were scanned with an Agilent DNA Microarray scanner (Agilent Technologies) at 5 µm resolution. The resulting images were analyzed by determining the Cy3 fluorescence intensity of all gene spots (features) on each array using the Agilent Feature Extraction Software (Version 10.7). The raw data were then uploaded into the ArrayTrack database [24]. The median fluorescence intensity of all the pixels within one feature was taken as the intensity value for that feature. The raw intensity values were then normalized using 75 percentile channel scaling normalization using ArrayTrack. A list of differentially expressed genes was generated with ArrayTrack using a *t*-test at p-value <0.05 and a fold change at >2.0.

### Functional analysis of significant genes

Ingenuity Pathway Analysis software (IPA, IPA version 9.0; Ingenuity Systems, Redwood City, CA, USA) was used to determine canonical pathways that were enriched for significant mRNA transcripts identified from the *t*-test analysis. Significance values were calculated based upon a right-tailed Fisher's exact test that determined whether a pathway was overrepresented by calculating whether the genes in a given pathway were enriched within the data set compared to all genes on the array; p<0.05 was selected as the cutoff for significance based on IPA threshold recommendations. Only those pathways with a p-value below the threshold and having more than three representative genes in the data set were considered significant.

### Quantitative reverse transcription-PCR (qRT-PCR)

Total RNA (2 µg) was reverse transcribed using random primers and High Capacity cDNA Reverse Transcription kit (Life Technologies, Grand Island, NY, USA) according to the manufacturer's protocol, and cDNA was analyzed in a 96-well plate assay format using the 7900HT Fast Real-Time PCR System (Life Technologies). Each plate contained experimental genes, and a housekeeping gene. All primers were obtained from Applied Biosystems. The cycle threshold (C_t_) for each sample was determined from the linear region of the amplification plot. The ΔC_t_ values for all genes were determined relative to the endogenous control glyceraldehyde 3-phosphate dehydrogenase (*Gapdh*). The ΔΔC_t_ values were calculated using treated group means relative to strain-matched control group means [25]. The fold change data were calculated from the ΔΔC_t_ values. All qRT-PCR reactions were conducted in triplicate and repeated twice.

### Western blot analysis

The level of trimethylation of histone H3 lysine 4 (H3K4), H3 lysine 9 (H3K9),H3 lysine 27 (H3K27), and H4 lysine 20 (H4K20), dimethylation of H3K9 and H4K20, acetylation of H3K9, H3K27, and H4 lysine 16 (H4K16), and the levels of tuberous sclerosis 2 (TSC2) protein and AKT in the cerebellum of BTBR T+tf/J and C57BL/6J mice was analyzed by Western blot analysis as described in Tryndyak *et al.* [26]. The following primary antibodies, rabbit polyclonal anti-H3K4me3 (Millipore, #07-473), anti-H3K9me3 (Millipore, #07-523), anti-H3K27me3 (Millipore, #07-449), and anti-H4K20me3 (Millipore, #07-463), anti-H3K9me2 (Millipore, #07-212), anti-H4K20me2 (Millipore, #07-367), anti-H3K9ac (Millipore, #06-942), anti-H3K27ac (Millipore, #07-360), anti-H4K16ac (Millipore, #06-762); rabbit monoclonal anti-Tuberin/TSC2 (D93F12) XP (Cell Signaling, #4308)), and anti-protein kinase B (AKT/pan) (C67E7) (Cell Signaling, #4691)) at 1∶1000 dilution were used for immunoblotting.

### Gene-specific DNA methylation

The methylation status of the mouse *Ogg1* promoter region was determined by direct bisulfite-sequencing analysis as previously described [27], [28].

### Statistical analyses

Results are presented as mean ± S.D. Data were analyzed by one-way analysis of variance (ANOVA), with pair-wise comparisons being made by the Student-Newman-Keuls method. When necessary, the data were natural log transformed before conducting the analyses to maintain a more equal variance or normal data distribution. Pearson product-moment correlation coefficients were used to determine the relationship between levels of gene expression and 8-oxodG. P-values<0.05 were considered significant.

## Results

### Microarray analysis of gene expression in the cerebellum of BTBR T+tf/J and C57BL/6J mice

**Figure 1:**
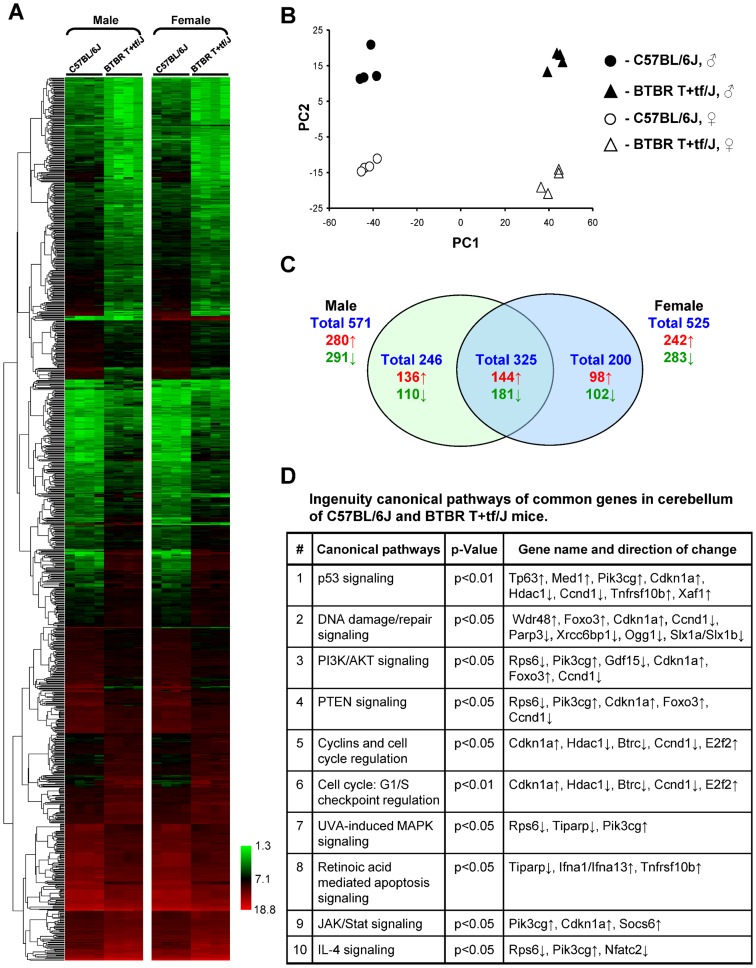
Whole genome microarray analysis of gene expression in the cerebellum of BTBR T+tf/J and C57BL/6J mice. (**A**) Heat map illustrating significant differences in global gene expression between BTBR T+tf/J and C57BL/6J mice. The color bar identifies high-expressed (red) and low-expressed (green) genes. (**B**) Principal component analysis illustrating similarities and differences between BTBR T+tf/J and C57BL/6J mouse strains. (**C**) Venn diagram showing genes that were significantly different between BTBR T+tf/J and C57BL/6J mice. (**D**) Summary of molecular pathways that significantly differ between BTBR T+tf/J and C57BL/6J mice. The Ingenuity Pathway Analysis database (version 9.0) was used to determine and visualize molecular pathways enriched by the significant mRNA transcripts a P-value of <0.05 were considered “enriched”.

In recent years a transcriptomic profiling has become a powerful tool for a better understanding of molecular pathways and uncovering the underlying mechanisms in the pathogenesis of various pathological states, including autism. Therefore, gene expression profiles in the cerebellum of BTBR T+tf/J and C57BL/6J mice were examined using high-throughput Agilent whole genome 8×60 K mouse microarrays. The microarray gene expression data have been deposited in the NCBI's Gene Expression Omnibus (GEO) database (accession number GSE62594). Unsupervised hierarchical clustering of the gene expression data showed that each mouse strain could be distinguished by its gene expression profile (Figure 1A). The tight clustering of samples within each mouse strain indicated the high quality data that would allow subtle differences in gene expression to be identified. Principal component analysis utilizing the entire gene expression dataset also showed the relatively tight clustering of each strain and the clear difference between gene expression profiles in the cerebellum of BTBR T+tf/J and C57BL/6J mice (Figure 1B).

To identify genes that were differentially expressed between BTBR T+tf/J and C57BL/6J mice, a t-test, p<0.05, coupled with a fold-change cut-off>2 was applied. A total of 325 genes, common for male and female mice, were found to be differentially expressed, up-regulated and down-regulated, in the cerebellum of BTBR T+tf/J mice as compared to C57BL/6J mice (Figure 1C). Despite the fact that differential expression of the majority of these genes may be driven by genomic variations between these mouse strains, a pathway enrichment analysis of the differentially expressed genes showed a strong enrichment in critical cell cycle/stress-related genes associated with DNA damage, chromatin organization, and cell death signaling, including the XIAP associated factor 1 (*Xaf1*), WD repeat domain 48 (*WD48*), forkhead box protein O3 (*Foxo3*), cyclin-dependent kinase inhibitor 1A (*Cdkn1a*, *p21*), cyclin D1 (*Ccnd1*), poly(ADP-ribose) polymerase 3 (*Parp3*), and *Ogg1* in the cerebellum of BTBR T+tf/J mice (Figure 1D and Table S1).

### Oxidative DNA damage in BTBR T+tf/J mice and human cerebellum

**Figure 2:**
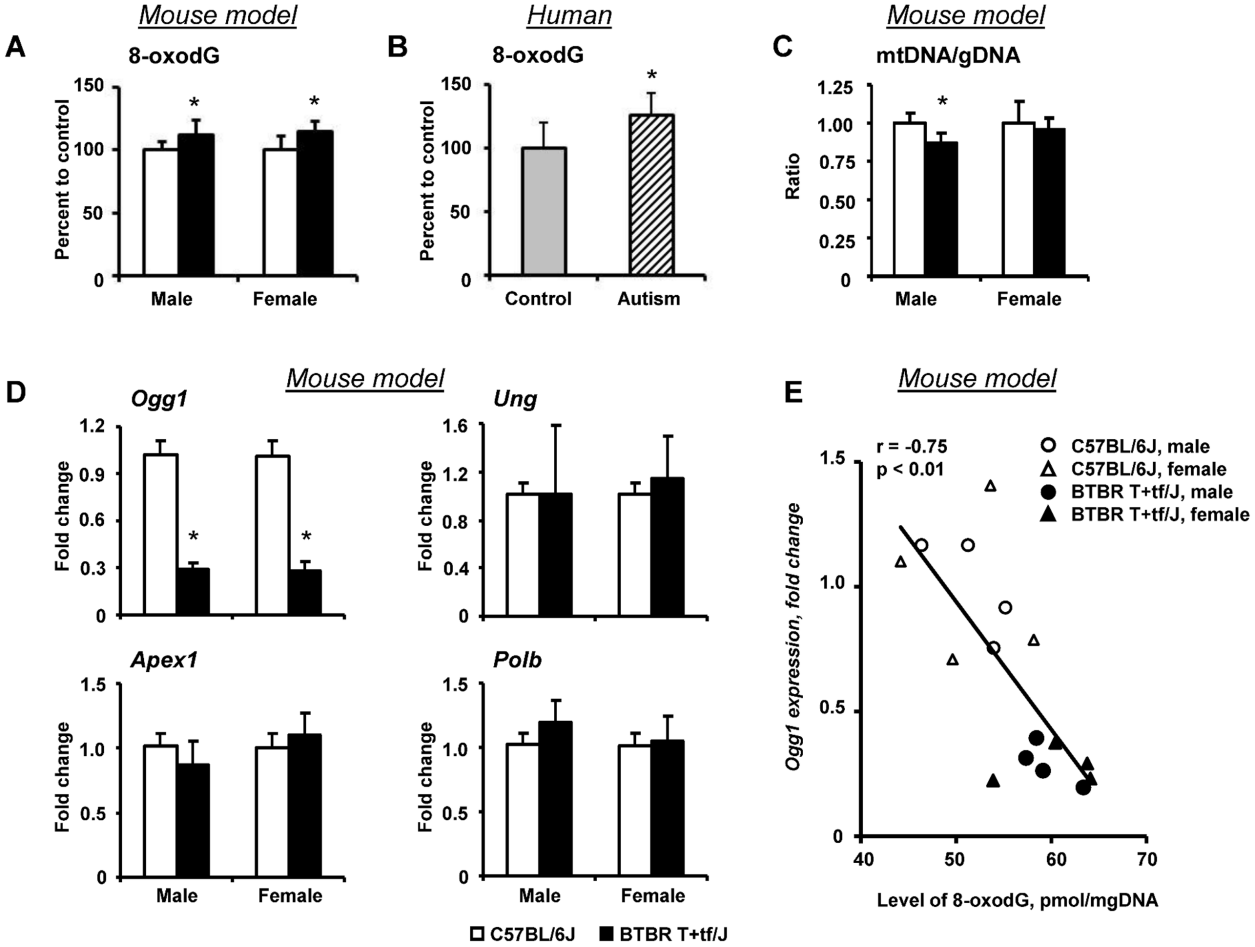
Oxidative DNA damage in the cerebellum of BTBR mice. (**A**) Levels of 8-oxo-7-hydrodeoxyguanosine (8-oxodG) in genomic DNA isolated from the cerebellum of BTBR T+tf/J and C57BL/6J mice (mean ± SD, n = 5). (**B**) Levels of 8-oxodG in genomic DNA isolated from the cerebellum of autism individuals and unaffected control individuals (mean ± SD, n = 13). (**C**) The extent of mitochondrial DNA damage in the cerebellum of BTBR T+tf/J mice (mean ± SD, n = 5). (**D**) The expression of base excision DNA repair genes in the cerebellum of BTBR T+tf/J and C57BL/6J mice. The expression of *Ogg1, Ung, Apex1*, and *Polβ* genes was determined by qRT-PCR as detailed in “Materials and Methods”. The results are presented as an average fold change in the expression of each gene in the cerebellum of BTBR T+tf/J mice relatively to that in C57BL/6J mice, which was assigned a value 1. * - Significantly different from C57BL/6J mice (mean ± SD, n = 5). (**E**) Correlation plots of the expression of *Ogg1* and the 8-oxodG level in mouse genomic DNA. Each symbol represents an individual animal.

The *Xaf1* gene was the most up-regulated gene in the cerebellum of male and female BTBR T+tf/J mice, with expression levels 238- and 211-times greater, respectively, than in C57BL/6J mice (Table S1). Significant increases were also observed in the p53 apoptotic and redox signaling network. Recently, Park *et al* [29] reported an association between up-regulation of *XAF1* and generation of reactive oxygen species, one of the key pathophysiological events in the molecular pathogenesis of autism [15]. Based on these findings, the level of 8-oxodG in genomic DNA, one of the prevalent and most studied form of oxidative DNA damage caused by reactive oxygen species [30], was assessed in the cerebellum of BTBR T+tf/J and C57BL/6J mice. Figure 2A shows that the level of 8-oxodG in genomic DNA in BTBR T+tf/J cerebellum was slightly, but significantly increased. We also evaluated the level of 8-oxodG in genomic DNA in postmortem cerebellum of individuals with autism, which was substantially greater than in unaffected control individuals (Figure 2B). In addition to the damage of genomic DNA, the compromise of mitochondrial functioning and integrity of mitochondrial DNA has been reported in individuals with autism [19], [31]. Because mitochondria are a major source as well as a target of free radical damage, the extent of mitochondrial DNA damage was evaluated by determining the mtDNA/nDNA ratio in the cerebellum of BTBR T+tf/J and C57BL/6J mice. Figure 2C shows that mtDNA/nDNA ratio in the cerebellum of male BTBR T+tf/J mice was significantly lower (p<0.05) as compared to male C57BL/6J mice, while it was not different in female BTBR T+tf/J and C57BL/6J mice.

### Expression of base excision DNA repair genes in BTBR T+tf/J mice

In order to investigate the potential mechanism for the persistence of 8-oxodG in genomic DNA in BTBR T+tf/J mice, the expression of key genes involved in the repair of oxidative DNA lesions was examined by qRT-PCR. The expression of base excision DNA repair genes has been shown to be a reliable biomarker of oxidative DNA damage [32]. Figure 2D shows a marked gender-independent decrease in the expression of *Ogg1* in the cerebellum of BTBR T+tf/J mice, while other three key genes in the base excision DNA repair pathway, uracil-DNA glycosylase (*Ung*), apurinic/apyrimidinic endonuclease 1 (*Apex1*), and DNA polymerase β (*Polβ*) did not differ from C57BL/6J mice. The expression of *Ogg1* in the cerebellum of male and female BTBR T+tf/J mice was 70% and 73% lower, respectively, than in age-matched C57BL/6J mice. More importantly, a decrease in the expression of *Ogg1* was significantly inversely correlated with the level of 8-oxodG in the cerebellum of BTBR T+tf/J mice (p<0.01) (Figure 2E).

### Status of DNA methylation in BTBR T+tf/J mice and human cerebellum and histone lysine modifications in BTBR T+tf/J mice

**Figure 3:**
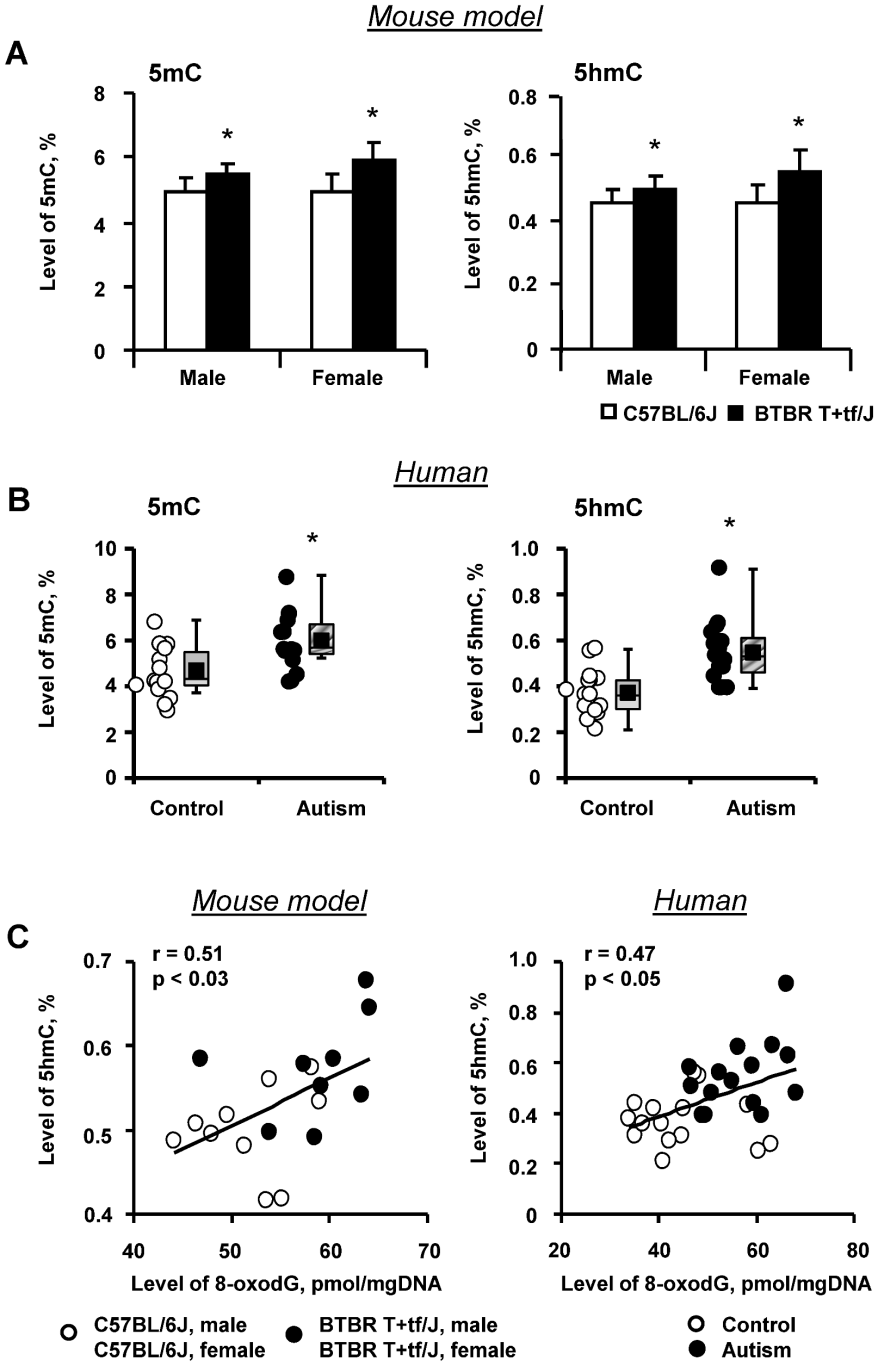
Levels of 5mC and 5hmC in cerebellar genomic DNA. (**A**) Levels of 5-methylcytosine (5mC) and 5-hydroxymethylcytosine (5hmC) in genomic DNA isolated from the cerebellum of BTBR T+tf/J and C57BL/6J mice (mean ± SD, n = 5). (**B**) Levels of 5mC and 5hmC in genomic DNA isolated from the cerebellum of autism individuals and unaffected control individuals. Values were represented as open and closed circles as well as box plots. * - Significantly different from C57BL/6J mice or autism-free individuals. (**C**) Correlation plots of the 5hmC and the 8-oxo-7-hydrodeoxyguanosine (8-oxodG) levels in mouse and human cerebellar genomic DNA.

**Figure 4:**
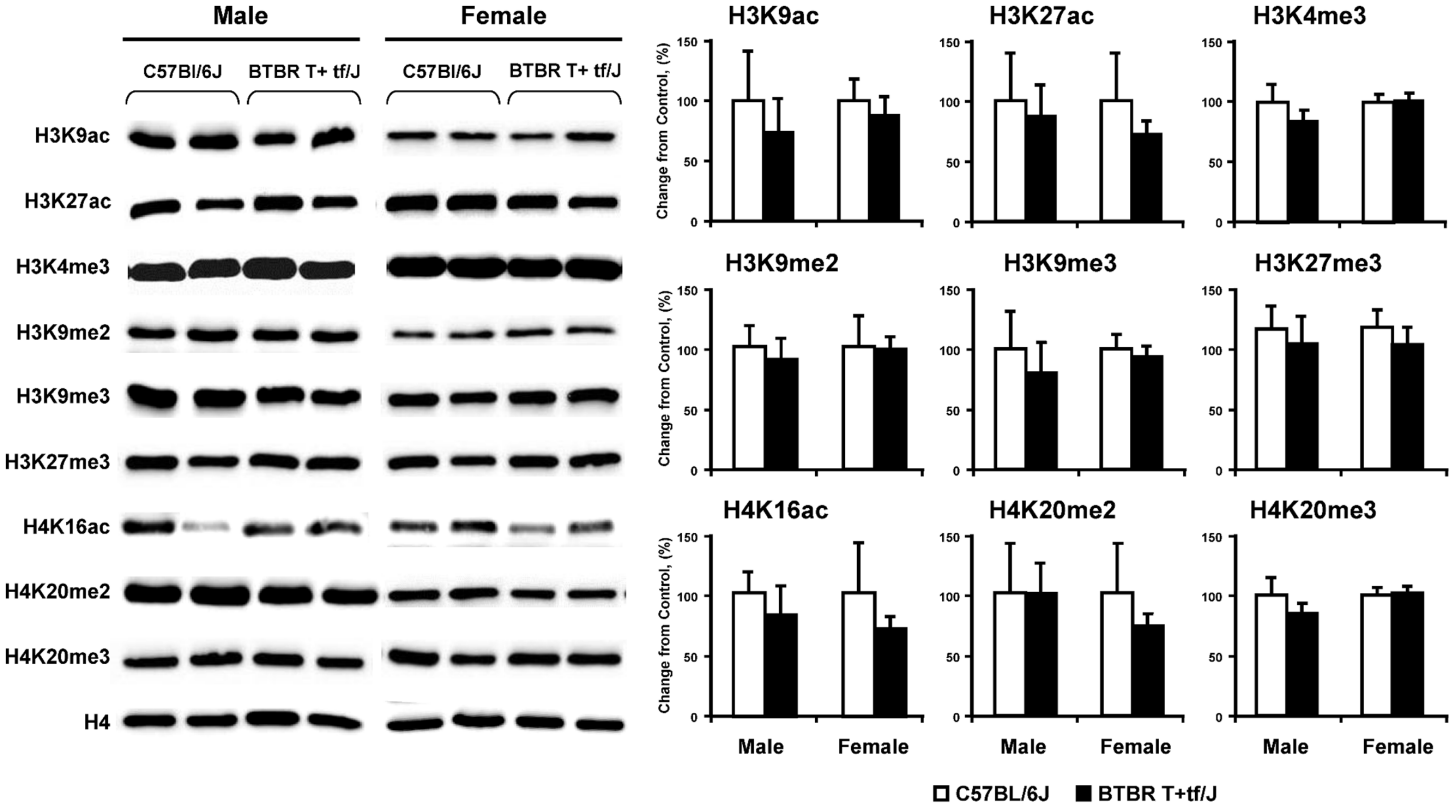
Western blot analysis of histone H3K4, H3K9, H3K27, and H4K20 trimethylation; H3K9, H4K20 dimethylation and H3K9, H3K27, and H4K16 acetylation in the cerebellum of BTBR T+tf/J and C57BL/6J mice. Densitometric analysis of the immunostaining results is shown as percent change in histone modification level in the cerebellum BTBR T+tf/J mice relative to the corresponding values in C57BL/6J mice, which was assigned a value of 100% (mean ± SD, n = 5).

**Figure 5:**
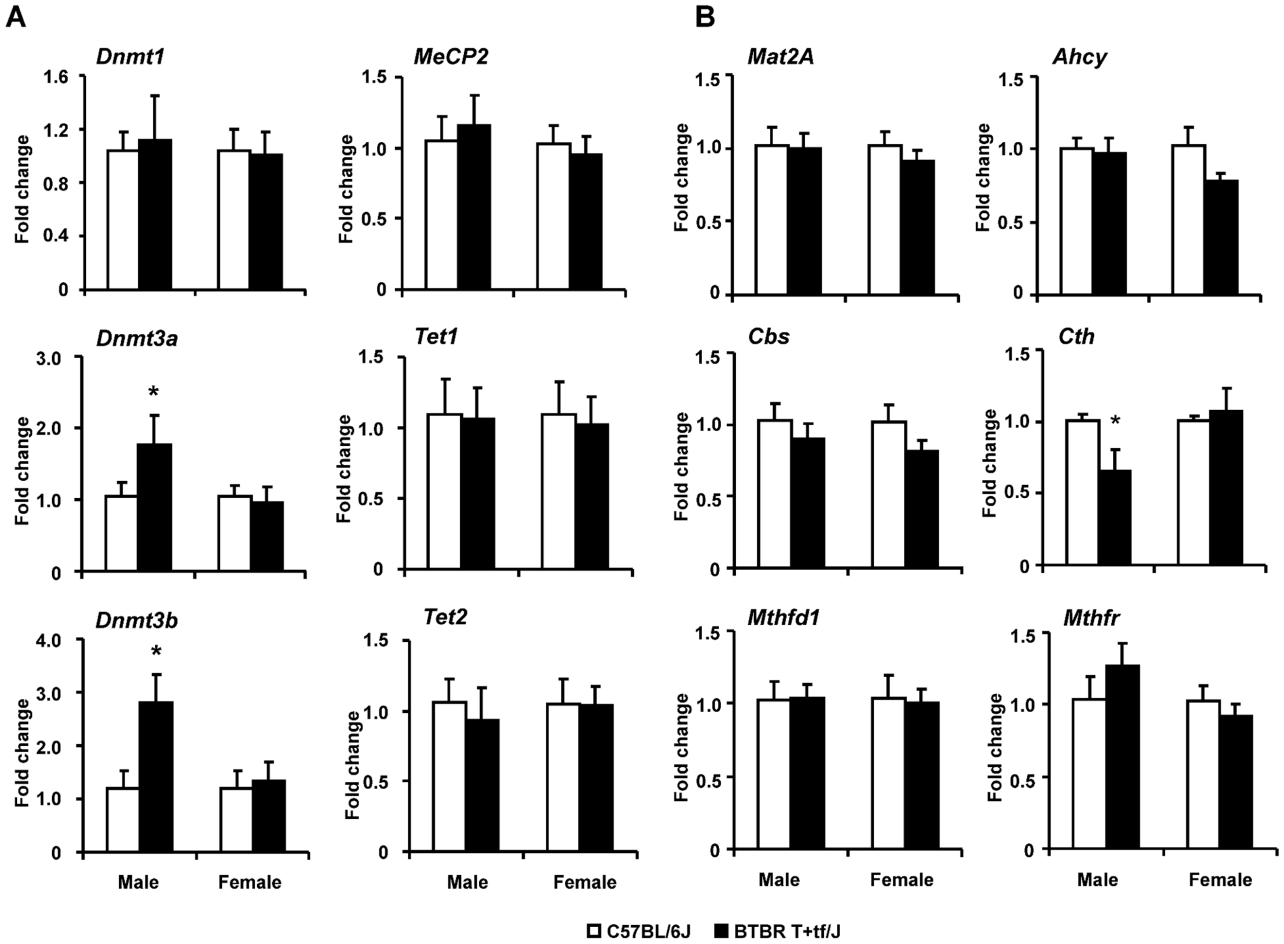
The expression of chromatin-modifying (A) and one carbon metabolism (B) genes in the cerebellum of BTBR T+tf/J and C57BL/6J mice. The gene expression was determined by qRT-PCR as detailed in “Materials and Methods”. The results are presented as an average fold change in the expression of each gene in the cerebellum of BTBR T+tf/J mice relatively to that in C57BL/6J mice, which was assigned a value 1. * - Significantly different from C57BL/6J mice (mean ± SD, n = 5).

It has been reported that one of the consequences of oxidative DNA damage is aberrant methylation of DNA [33], [34]. Therefore, the status of global DNA methylation in the cerebellum of BTBR T+tf/J and C57BL/6J mice was evaluated. Because traditional methods do not distinguish between 5mC and 5hmC, we evaluated both forms of methylated cytosines independently. Figure 3A shows that the levels of 5mC and 5hmC in genomic DNA were significantly increased in the cerebellum of both male and female BTBR T+tf/J mice. Importantly, the level of 5mC and 5hmC elevation in genomic DNA in the postmortem human cerebellum of individuals with autism was similarly increased as compared to matched unaffected control individuals (Figure 3B) with a magnitude substantially greater than in BTBR T+tf/J mice. Importantly, the level of 5hmC in both mouse and human cerebellar genomic DNA was significantly correlated with the extent of 8-oxodG accumulation in DNA (Figure 3C). In addition to alterations in DNA methylation, the disruption of a normal pattern of covalent histone modifications is another epigenetic change frequently found in human neurodevelopmental diseases, including autism [35]. Hence, the levels of H3K4me3, H3K9me3, H3K27me3, H4K20me3, H3K9ac, and H3K56ac in the cerebellum of BTBR T+tf/J and C57BL/6J mice were evaluated (Figure 4). It is well established that these histone lysine modifications play a crucial role in the maintenance of genomic stability, chromatin assembly and organization, DNA damage and repair, and regulation of gene transcription [36], [37]. Figure 5 shows that in contrast to DNA methylation changes, the levels of selective histone lysine methylation and acetylation marks in the cerebellum of BTBR T+tf/J were not different from that in C57BL/6J mice.

### Expression of chromatin-modifying and one-carbon metabolism genes in BTBR T+tf/J mice

The status of DNA methylation and histone modifications relies on several factors, especially on the expression and functioning of chromatin modifying genes and proteins, availability of methyl-group donors and the functioning of one-carbon metabolism, and integrity of DNA [35]. Figure 5A shows that the expression of the *de novo* DNA methyltransferases, *Dnmt3a* and *Dnmt3b*, was, respectively, 1.5- and 1.8-times greater in the cerebellum of in male BTBR T+tf/J mice than in C57BL/6J mice. The expression of *Dnmt1*, *MeCP2*, *Tet1*, and *Tet2* (Figure 4A) and histone modifying genes (Figure S1) in the cerebellum of BTBR T+tf/J mice did not differ from those in C57BL/6J mice.

The expression of one-carbon metabolism genes did not differ between mouse strains except for the expression of an essential trans-sulfuration pathway gene, cystathionine γ-lyase (*Cth*), in which inhibition is associated with a greater vulnerability to oxidative injury [38] (Figure 5B). The expression of *Cth* was reduced by 44% in male BTBR T+tf/J mice as compared to C57BL/6J mice.

### Mechanism of Ogg1 down-regulation in BTBR T+tf/J mice

The expression of *Ogg1* may be compromised by genetic factors, e.g., single nucleotide polymorphisms, epigenetic factors, e.g., promoter methylation, and an altered function of its transcription regulators, e.g., TSC2. Figure S2-A shows that the *Ogg1* gene in BTBR T+tf/J mice is characterized by the presence of four amino acid coding single nucleotide polymorphisms, three in exon 2 and one in exon 3, as compared to C57BL/6J mice. In contrast, a bisulfite sequencing analysis of the *Ogg1* promoter region shows no difference in cytosine methylation between BTBR T+tf/J and C57BL/6J mice (Figure S2-B). Likewise, western blot analysis revealed no difference in the levels of TSC2 and AKT proteins in the cerebellum in these two mouse strains (Figure S2-C).

## Discussion

Autism is a complex disorder that is thought to be the consequence of multiple interdependent events during development [3], [4], [6]. The transcriptomic analysis of the cerebellum of BTBR T+tf/J mice demonstrates a profoundly dysregulated expression of cell cycle/stress-related genes, mainly p53 apoptotic signaling, DNA damage and repair, and chromatin modifying genes. These findings are in good agreement with previous reports that have convincingly established a dysregulation of these molecular pathways in autistic brain [4], [15], [16], [39]. Oxidative stress-induced damage to DNA and aberrant DNA methylation are considered as key events, since both of them, in addition to genetic factors, may contribute to the heritability of autism. In this study, we demonstrate that DNA isolated from the cerebellum of BTBR T+tf/J mice and post-mortem cerebellum of individuals with autism, is characterized by an increased levels of 8-oxodeoxyguanosine, 5-methylcytosine, and 5-hydroxymethylcytosine.

8-oxodG is one of the prevalent and most studied oxidative DNA lesion and oxidative stress marker [30]. Several comprehensive studies have established the important role of the altered cellular redox status in the pathophysiology of autism [15]. The majority of those studies have largely focused on the potential causes of the oxidative stress induction, mainly on the glutathione redox imbalance and mitochondrial dysfunction [17]–[22]. In contrast, the underlying mechanisms associated with the persistence of oxidative lesions in DNA and proteins in the pathogenesis of autism have never been addressed completely beyond the establishing their presence in autistic brain [40]. The results of the present study demonstrate that an increase in the level of 8-oxodG in the cerebellum of BTBR T+tf/J mice may be largely related to the profound inhibition of the *Ogg1* expression. In addition to *Ogg1* down-regulation, an observed up-regulation of *Cdkn1a* and *Ccnd1* genes may contribute to an elevation of 8-oxodG in DNA in BTBR T+tf/J mice [41], [42].

OGG1 is a key enzyme preventing the accumulation of 8-oxodG in DNA through recognizing and removing 8-oxodG from DNA and initiating the highly conserved base excision repair pathway [43]. Since OGG1 is the first enzyme in the base excision DNA repair pathway, the accurate DNA repair greatly depends on the ability of OGG1 to remove 8-oxodG. *Ogg1* is highly expressed in the brain and has been shown to protect neurons against oxidative DNA damage during development and various pathologic conditions [44], [45]. A lack of *Ogg1* in the brain resulted in multiple cellular and molecular events, including increased apoptosis and aberrant neuronal connectivity [45], key pathomorphological features of autism. Additionally, it is well-established that the accumulation of 8-oxodG in the genome caused by inhibition of OGG1 is a key event in the pathogenesis of several human pathologies, including cancer [46], neurodegeneration [47], Parkinson's disease [48], and obesity and metabolic dysfunction [49].

Several mechanisms may contribute to the inhibition of *Ogg1* expression in BTBR T+tf/J mice. Since the promoter region of the mouse *Ogg1* gene contains a strong CpG island and because of an elevated genomic content of 5mC, a number of epigenetic mechanisms may be involved in *Ogg1* gene silencing. However, the bisulfite sequencing analysis of the *Ogg1* promoter region did not show differences in CpG methylation between the two mouse strains. It is possible that gene-specific histone modifications at the *Ogg1* promoter region could cause an inhibition of the *Ogg1* expression in the cerebellum of BTBR T+tf/J mice; however, despite the fact that we did not observe alterations in the level of global histone modifications. There are other interconnected molecular mechanisms that could contribute to *Ogg1* down-regulation. Specifically, it is well-established that TSC2 is a key regulator of the *Ogg1* gene [50]. Down-regulation of TSC2 or genetic deficiency of *Tsc2* has been reported to cause a marked decrease of *Ogg1* mRNA that was accompanied by the accumulation of 8-oxoG in DNA [50]. The loss of TSC2 has been associated with various neuropsychological disorders [51]. Importantly Reith *et al.* [52], [53] and Tsai *et al.* [54] have reported that genetic *Tsc2* or *Tsc1* deficiency causes Purkinje cell degeneration and the development of autism-like phenotype. The results of the present study demonstrating only moderate changes in the level of TSC2 protein in the cerebellum of BTBR T+tf/J mice indicate that this mechanism may not be a main cause of the *Ogg1* inhibition. Finally, inhibition of *Ogg1* may be caused by genetic variations. A computational analysis of the *Ogg1* gene, using the GeneNetwork database (www.genenetwork.org), revealed substantial differences in single nucleotide polymorphisms located in coding and non-coding regions of *Ogg1* between BTBR T+tf/J and C57BL/6J mice that may explain a reduced gene expression in BTBR T+tf/J mice.

Another important finding of the present study is a substantial increase in the levels of 5mC and 5hmC in the cerebellum of BTBR T+tf/J mice that is also present in the cerebellum of individuals with autism [16], [55]. The increased level of 5mC in the cerebellum of BTBR T+tf/J mice found in this study may be attributed to (i) the presence of oxidative DNA lesions [34] and (ii) a marked up-regulation of *de novo* DNA methyltransferases *Dnmt3a* and *Dnmt3b* (Figure 5). Many current reports link the increase in 5hmC content in DNA to the demethylating function of TET enzymatic oxidation of 5mC [56]. The results of the present study demonstrate that an increased level of 5hmC in the cerebellum of BTBR T+tf/J mice occurred without changes in the *Tet1* and *Tet2* expression. This corresponds to similar findings in the mouse hippocampus during aging [57]. A parallel elevation of 5mC and 5hmC in DNA in the cerebellum of BTBR T+tf/J mice and individuals with autism suggest that the increase in 5hmC level is not due to its role as an intermediate during demethylation of DNA. A significant positive correlation between the 8-oxoG and 5hmC in both mouse and human cerebellum suggests that the mechanism of DNA methylation alterations found in this study may be a consequence of an altered cellular redox status and oxidative stress.

In conclusion, the results of our study demonstrate that oxidative DNA lesions and an altered pattern of DNA methylation are important molecular features of the autism cerebellar phenotype. The data presented herein point that diminished expression of *Ogg1* in the cerebellum of BTBR T+tf/J mice caused by single nucleotide variation in the *Ogg1* gene might be a driving force that promotes the accumulation of 8-oxodG in DNA. Similar alterations in 8-oxodG in cerebellar tissue from humans with autism and BTBR T+tf/J mouse model warrant future large-scale studies to specifically address the role genetic alterations *OGG1* in pathogenesis of autism in human population.

## Supporting Information

Figure S1 **The expression of histone-modifying genes in the cerebellum of BTBR T+tf/J and C57BL/6J mice.** The gene expression was determined by qRT-PCR as detailed in “Materials and Methods”. The results are presented as an average fold change in the expression of each gene in the cerebellum of BTBR T+tf/J mice relatively to that in C57BL/6J mice, which was assigned a value 1 (mean ± SD, n = 5).
(TIF)

Figure S2 **Potential mechanisms of** ***Ogg1*** **down-regulation in the cerebellum of BTBR T+tf/J mice.** (**A**) A Diagram showing single nucleotide polymorphism differences in the coding region of the *Ogg1* gene in BTBR T+tf/J and C57BL/6J mice. (**B**) Bisulfite sequencing analysis of the *Ogg1* promoter methylation. (**C**) Western blot analysis of TSC2 and AKT in the cerebellum of BTBR T+tf/J and C57BL/6J mice.
(TIF)

Table S1 Common differentially expressed genes in the cerebellum of BTBRT+tf/J and C57BL/6J mice. (n = 4).
(DOC)

## References

[1] Shstry BS (2003) Molecular genetics of autism spectrum disorders. Journal of Human Genetics 48:495–501. 13680297 10.1007/s10038-003-0064-9

[2] Betancur C (2011) Etiological heterogeneity in autism spectrum disorders: more than 100 genetic and genomic disorders and still counting. Brain Research 1380:42–77. 21129364 10.1016/j.brainres.2010.11.078

[3] Persico AM, Napolioni V (2013) Autism genetics. Behavioural Brain Research 251:95–112. 23769996 10.1016/j.bbr.2013.06.012

[4] Rossignol DA, Frye RE (2012) A review of research trends in physiological abnormalities in autism spectrum disorders: immune dysregulation, inflammation, oxidative stress, mitochondrial dysfunction and environmental toxicant exposures. Molecular Psychiatry 17:389–401. 22143005 10.1038/mp.2011.165 PMC3317062

[5] Fatemi SH, Aldinger KA, Ashwood P, Bauman ML, Blaha CD, et al (2012) Consensus paper: pathological role of the cerebellum in autism. Cerebellum 11:777–807. 22370873 10.1007/s12311-012-0355-9 PMC3677555

[6] Wang SS-H, Kloth AD, Badura A (2014) The cerebellum, sensitive periods, and autism. Nueron 83:518–532. 10.1016/j.neuron.2014.07.016 PMC4135479 25102558

[7] Becker EB, Stoodley CJ (2013) Autism spectrum disorder and the cerebellum. International Review of Neurobiology 113:1–34. 24290381 10.1016/B978-0-12-418700-9.00001-0

[8] Allen G, Courchesne E (2003) Differential effects of developmental cerebellar abnormality on cognitive and motor functions in the cerebellum: an fMRI study of autism. The American Journal of Psychiatry 160:262–273. 12562572 10.1176/appi.ajp.160.2.262

[9] Moy SS, Nadler JJ, Young NB, Perez A, Holloway LP, et al (2007) Mouse behavioral tasks relevant to autism: phenotypes of ten inbred strains. Behavioural Brain Research 176:4–20. 16971002 10.1016/j.bbr.2006.07.030 PMC1857288

[10] McFarlane HG, Kusek GK, Yang M, Phoenix JL, Bolivar VJ, et al (2008) Autism-like behavioral phenotypes in BTBR T+tf/J mice. Genes, Brain and Behavior 7:152–163. 10.1111/j.1601-183X.2007.00330.x 17559418

[11] Chadman KK, Guariglia SR (2012) The BTBR T+tf/J (BTBR) mouse model of autism. Autism S1:009.

[12] Meyza KZ, Defensor EB, Jensen AL, Corley MJ, Pearson BL, et al (2013) The BTBR T+ tf/J mouse model for autism spectrum disorders-in search of biomarkers. Behavioural Brain Research 251:25–34. 22958973 10.1016/j.bbr.2012.07.021 PMC3529977

[13] Onore CE, Careaga M, Babineau BA, Schwartzer JJ, Berman RF, et al (2013) Inflammatory macrophage phenotype in BTBR T+tf/J mice. Frontiers in Neuroscience 7:158. 24062633 10.3389/fnins.2013.00158 PMC3774991

[14] Dodero L, Damiano M, Galbusera A, Bifone A, Tsaftsaris SA, et al (2013) Neuroimaging evidence of major morpho-anatomical and functional abnormalities in the BTBR T+TF/J mouse model of autism. PLoS One 8:e76655. 24146902 10.1371/journal.pone.0076655 PMC3797833

[15] Frustaci A, Neri M, Cesario A, Adams JB, Domenici E, et al (2012) Oxidative stress-related biomarkers in autism: systematic review and meta-analyses. Free Radical Biology & Medicine 52:2128–2141. 22542447 10.1016/j.freeradbiomed.2012.03.011

[16] Ladd-Acosta C, Hansen KD, Briem E, Fallin MD, Kaufmann WE, et al (2014) Common DNA methylation alterations in multiple brain regions in autism. Molecular Psychiatry 19:862–71. 23999529 10.1038/mp.2013.114 PMC4184909

[17] James SJ, Rose S, Melnyk S, Jernigan S, Blossom S, et al (2009) Cellular and mitochondrial glutathione redox imbalance in lymphoblastoid cells derived from children with autism. The FASEB Journal 23:2374–2383. 19307255 10.1096/fj.08-128926 PMC2717775

[18] Bowers K, Li Q, Bressler J, Avramopoulos D, Newschaffer C, et al (2011) Glutathione pathway gene variation and risk of autism spectrum disorders. Journal of Neurodevelopmental Disorders 3:132–143. 21484198 10.1007/s11689-011-9077-4 PMC3188290

[19] Main PA, Angley MT, O'Doherty CE, Thomas P, Fenech M (2012) The potential role of the antioxidant and detoxification properties of glutathione in autism spectrum disorders: a systematic review and meta-analysis. Nutrition & Metabolism (Lond) 9:35. 10.1186/1743-7075-9-35 PMC3373368 22524510

[20] Chauhan A, Audhya T, Chauhan V (2012) Brain region-specific glutathione redox imbalance in autism. Neurochemical Research 37:1681–1689. 22528835 10.1007/s11064-012-0775-4

[21] Schaevitz LR, Berger-Sweeney JE (2012) Gene-environment interactions and epigenetic pathways in autism: the importance of one-carbon metabolism. ILAR Journal 53:322–340. 23744970 10.1093/ilar.53.3-4.322

[22] Rose S, Melnyk S, Pavliv O, Bai S, Nick TG, et al (2012) Evidence of oxidative damage and inflammation associated with low glutathione redox status in the autism brain. Translational Psychiatry 2:e134. 22781167 10.1038/tp.2012.61 PMC3410618

[23] James SJ, Shpyleva S, Melnyk S, Pavliv O, Pogribny IP (2013) Complex epigenetic regulation of engrailed-2 (EN-2) homeobox gene in the autism cerebellum. Translational Psychiatry 3:e232. 23423141 10.1038/tp.2013.8 PMC3590998

[24] Fang H, Harris SC, Su Z, Chen M, Qian F, et al (2009) ArrayTrack: an FDA and public genomic tool. Methods in Molecular Biology 563:379–398. 19597796 10.1007/978-1-60761-175-2_20

[25] Schmittgen TD, Livak KJ (2008) Analyzing real-time PCR data by the comparative C(T) method. Nature Protocols 3:1101–1108. 18546601 10.1038/nprot.2008.73

[26] Tryndyak VP, Muskhelishvili L, Kovalchuk O, Rodriguez-Juarez R, Montgomery B, et al (2006) Effect of long-term tamoxifen exposure on genotoxic and epigenetic changes in rat liver: implications for tamoxifen-induced hepatocarcinogenesis. Carcinogenesis 27:1713–1720. 16632870 10.1093/carcin/bgl050

[27] Jiang M, Zhang Y, Fei J, Chang X, Fan W, et al (2010) Rapid quantification of DNA methylation by measuring relative peak heights in direct bisulfite-PCR sequencing traces. Laboratory Investigation 90:282–290. 20010852 10.1038/labinvest.2009.132

[28] Parrish RR, Day JJ, Lubin FD (2012) Direct bisulfite sequencing for examination of DNA methylation with gene and nucleotide resolution from brain tissues. Current Protocols in Neuroscience Chapter 7: Unit 7.24. 10.1002/0471142301.ns0724s60 PMC3395468 22752896

[29] Park GB, Choi Y, Kim YS, Lee HK, Kim D, et al (2013) ROS and ERK1/2-mediated caspase-9 activation increases XAF1 expression in dexamethasone-induced apoptosis of EBV-transformed B cells. International Journal of Oncology 43:29–38. 23685456 10.3892/ijo.2013.1949 PMC3742161

[30] Dizdaroglu M, Jaruga P, Birincioglu M, Rodriguez H (2002) Free radical-induced damage to DNA: mechanisms and measurement. Free Radical Biology & Medicine 32:1102–1115. 12031895 10.1016/s0891-5849(02)00826-2

[31] Napoli E, Wong S, Giulivi C (2013) Evidence of reactive oxygen species-mediated damage to mitochondrial DNA in children with typical autism. Molecular Autism 4:2. 23347615 10.1186/2040-2392-4-2 PMC3570390

[32] Powell CL, Swenberg JA, Rusyn I (2005) Expression of base excision DNA repair genes as a biomarker of oxidative DNA damage. Cancer Letters 229:1–11. 16157213 10.1016/j.canlet.2004.12.002

[33] Valinluck V, Tsai HH, Rogstad DK, Burdzy A, Bird A, et al (2004) Oxidative damage to methyl-CpG sequences inhibits the binding of the methyl-CpG binding domain (MBD) of methyl-CpG binding protein 2 (MeCP2). Nucleic Acids Research 32:4100–4108. 15302911 10.1093/nar/gkh739 PMC514367

[34] O'Hagan HM, Wang W, Sen S, Destefano Shields C, Lee SS, et al (2011) Oxidative damage targets complexes containing DNA methyltransferases, SIRT1, and polycomb members to promoter CpG Islands. Cancer Cell 20:606–619. 22094255 10.1016/j.ccr.2011.09.012 PMC3220885

[35] Miyake K, Hirasawa T, Koide T, Kubota T (2012) Epigenetics in autism and other neurodevelopmental diseases. Advances in Experimental Medicine and Biology 724:91–98. 22411236 10.1007/978-1-4614-0653-2_7

[36] Jenuwein T (2006) The epigenetic magic of histone methylation. Federation of European Biochemical Societies Journal 273:3121–3135. 16857008 10.1111/j.1742-4658.2006.05343.x

[37] Ernst J, Kheradpour P, Mikkelsen TS, Shoresh N, Ward LD, et al (2011) Mapping and analysis of chromatine state dynamics in nine human cell types. Nature 473:43–51. 21441907 10.1038/nature09906 PMC3088773

[38] Ishii I, Akahoshi N, Yamada H, Nakano S, Izumi T, et al (2010) Cystathionine γ-lyase-deficient mice require dietary cysteine to protect against acute lethal myopathy and oxidative injury. Journal of Biological Chemistry 285:26358–26368. 20566639 10.1074/jbc.M110.147439 PMC2924062

[39] Araghi-Niknam M, Fatemi SH (2003) Levels of Bcl-2 and P53 are altered in superior frontal and cerebellar cortices of autistic subjects. Cellular and Molecular Neurobiology 23:945–952. 14964781 10.1023/B:CEMN.0000005322.27203.73 PMC11530152

[40] Sajdel-Sulkowska EM, Xu M, Koibuchi N (2009) Increase in cerebellar neurotrophin-3 and oxidative stress markers in autism. Cerebellum 8:366–372. 19357934 10.1007/s12311-009-0105-9

[41] Kinoshita A, Wanibuchi H, Imaoka S, Ogawa M, Masuda C, et al (2002) Formation of 8-hydroxydeoxyguanosine and cell-cycle arrest in the rat liver via generation of oxidative stress by phenobarbital: association with expression profiles of p21(WAF1/Cip1), cyclin D1 and Ogg1. Carcinogenesis 23:341–349. 11872643 10.1093/carcin/23.2.341

[42] Hyun JW, Yoon SH, Yu Y, Han CS, Park JS, et al (2006) Oh8dG induces G1 arrest in a human acute leukemia cell line by upregulating P21 and blocking the RAS to ERK signaling pathway. International Journal of Cancer 118:302–309. 16052517 10.1002/ijc.21329

[43] Klungland A, Bjelland S (2007) Oxidative damage to purines in DNA: role of mammalian Ogg1. DNA Repair (Amsterdam) 6:481–488. 10.1016/j.dnarep.2006.10.012 17127104

[44] Liu D, Croteau DL, Souza-Pinto N, Pitta M, Tian J, et al (2011) Evidence that OGG1 glycosylase protects neurons against oxidative DNA damage and cell death under ischemic conditions. Journal of Cerebral Blood Flow & Metabolism 31:680–692. 20736962 10.1038/jcbfm.2010.147 PMC3049522

[45] Wong AW, McGallum GP, Jeng W, Wells PG (2008) Oxoguanine glycosylase 1 protects against methamphetamine-enhanced fetal brain oxidative DNA damage and neurodevelopmental deficits. The Journal of Neuroscience 28:9054–9047. 10.1523/JNEUROSCI.2557-08.2008 PMC6670872 18768699

[46] Hirano T (2008) Repair system of 7, 8-dihydro-8-oxoguanine as a defense line against carcinogenesis. Journal of Radiation Research 49:329–340. 18596371 10.1269/jrr.08049

[47] Sheng Z, Oka S, Tsuchimoto D, Abolhassani N, Nomaru H, et al (2012) 8-Oxoguanine causes neurodegeneration during MUTYH-mediated DNA base excision repair. Journal of Clinical Investigation 12:4344–4361. 10.1172/JCI65053 PMC3533558 23143307

[48] Nakabeppu Y, Tsuchimoto D, Yamaguchi H, Sakumi K (2007) Oxidative damage in nucleic acids and Parkinson's disease. Journal of Neuroscience Research 85:919–934. 17279544 10.1002/jnr.21191

[49] Sampath H, Vartanian V, Rollins MR, Sakumi K, Nakabeppu Y, et al (2012) 8-Oxoguanine DNA glycosylase (OGG1) deficiency increases susceptibility to obesity and metabolic dysfunction. PLoS One 7:e51697. 23284747 10.1371/journal.pone.0051697 PMC3524114

[50] Habib SL, Riley DJ, Mahimainathan L, Bhandari B, Choudhury GG, et al (2008) Tuberin regulates the DNA repair enzyme OGG1. The American Journal of Physiology: Renal Physiology 294:F281–290. 17989114 10.1152/ajprenal.00370.2007

[51] Crino PB (2013) Evolving neurobiology of tuberous sclerosis complex. Acta Neuropathologica 125:317–332. 23386324 10.1007/s00401-013-1085-x

[52] Reith RM, Way S, McKenna J 3rd, Haines K, Gambello MJ (2011) Loss of the tuberous sclerosis complex protein tuberin causes Purkinje cell degeneration. Neurobiology of Disease 43:113–122. 21419848 10.1016/j.nbd.2011.02.014 PMC3096682

[53] Reith RM, McKenna J, Wu H, Hashmi SS, Cho SH, et al (2013) Loss of Tsc2 in Purkinje cells is associated with autistic-like behavior in a mouse model of tuberous sclerosis complex. Neurobiology of Disease 51:93–103. 23123587 10.1016/j.nbd.2012.10.014

[54] Tsai PT, Hull C, Chu Y, Greene-Colozzi E, Sadowski AR, et al (2012) Autistic-like behaviour and cerebellar dysfunction in Purkinje cell Tsc1 mutant mice. Nature 48:647–651. 10.1038/nature11310 PMC3615424 22763451

[55] James SJ, Shpyleva S, Melnyk S, Pavliv O, Pogribny IP (2014) Elevated 5-hydroxymethylcytosine in the Engrailed-2 (EN-2) promoter is associated with increased gene expression and decreased MeCP2 binding in autism cerebellum. Translational Psychiatry 4:e460. 25290267 10.1038/tp.2014.87 PMC4350522

[56] Piccolo FM, Fisher AG (2014) Getting rid of DNA methylation. Trends Cell Biology 24:136–43. 10.1016/j.tcb.2013.09.001 24119665

[57] Chen H, Dzitoyeva S, Manev H (2012) Effect of aging on 5-hydroxymethylcytosine in the mouse hippocampus. Restorative Neurology and Neuroscience 30:237–245. 22426040 10.3233/RNN-2012-110223 PMC3361533

