# Supplementary figures and images for: Cerebellar Oxidative DNA Damage and Altered DNA Methylation in the BTBR T+tf/J Mouse Model of Autism and Similarities with Human Post Mortem Cerebellum
Source: PLoS One. 2014 Nov 25;9(11):e113712. doi: 10.1371/journal.pone.0113712 (PMC4244134; doi:10.1371/journal.pone.0113712)

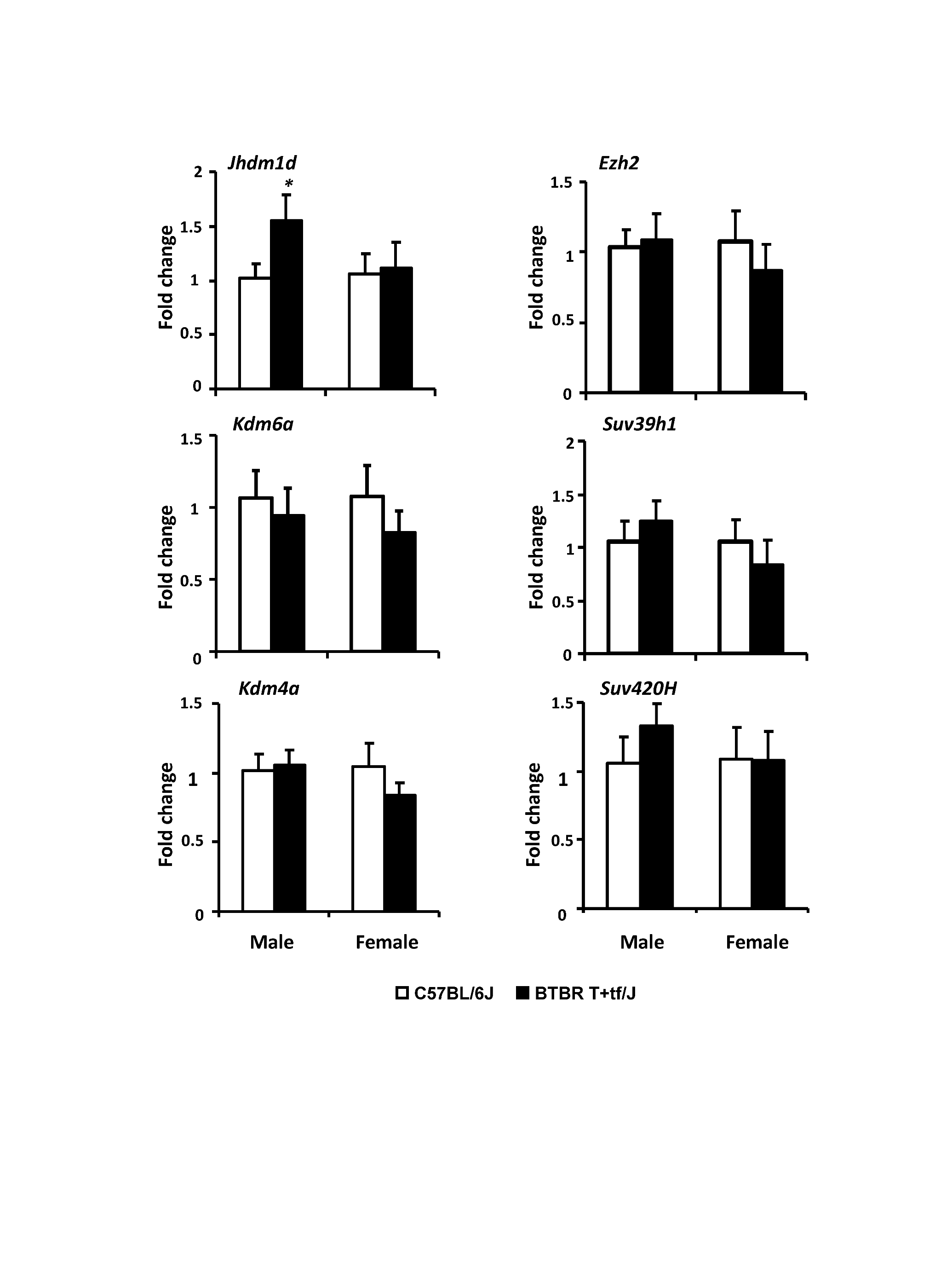

Supplement: Figure S1 — The expression of histone-modifying genes in the cerebellum of BTBR T+tf/J and C57BL/6J mice. The gene expression was determined by qRT-PCR as detailed in “Materials and Methods”. The results are presented as an average fold change in the expression of each gene in the cerebellum of BTBR T+tf/J mice relatively to that in C57BL/6J mice, which was assigned a value 1 (mean ± SD, n = 5). (TIF) [file pone.0113712.s001.tif]

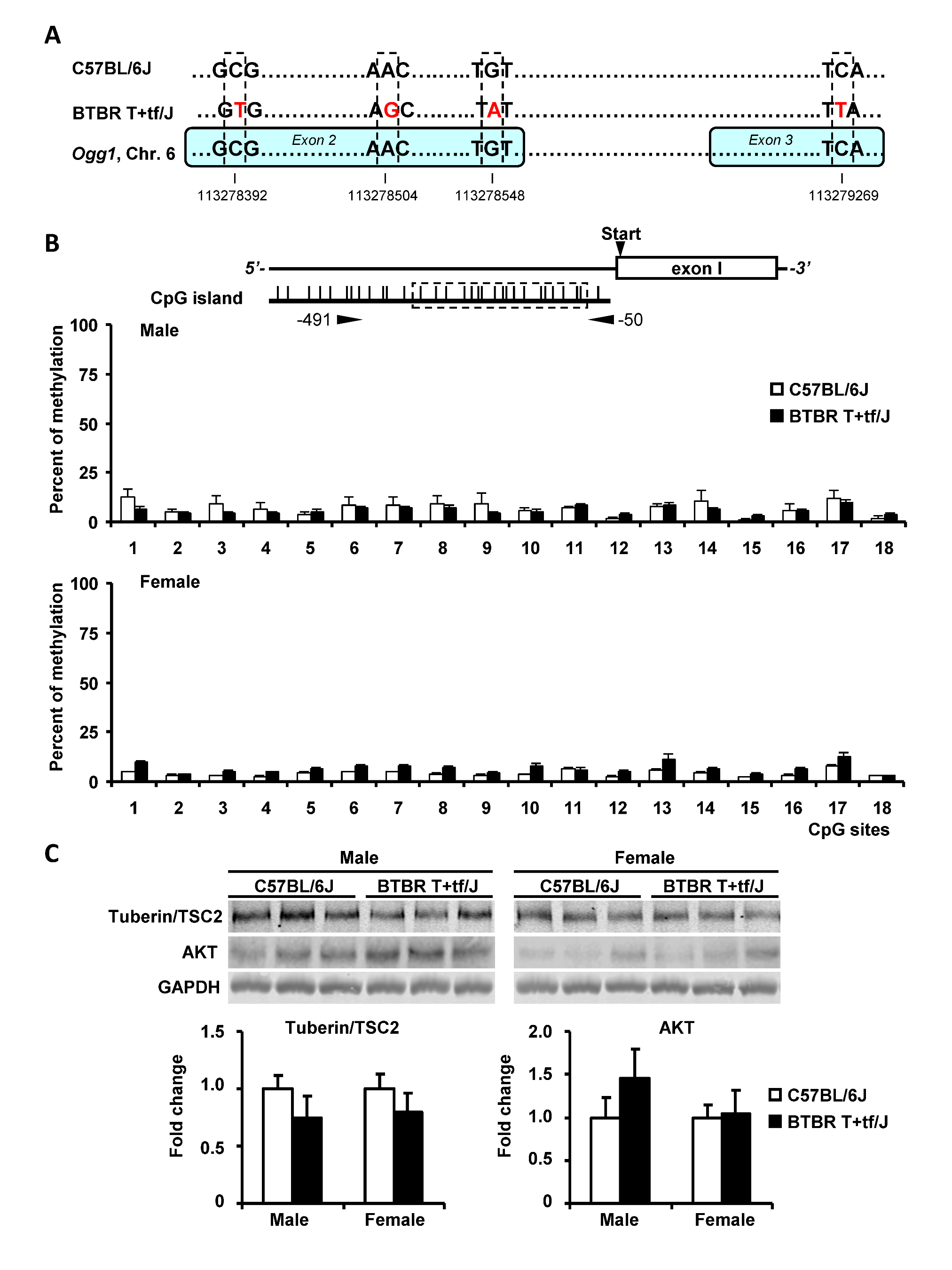

Supplement: Figure S2 — Potential mechanisms of Ogg1 down-regulation in the cerebellum of BTBR T+tf/J mice. (A) A Diagram showing single nucleotide polymorphism differences in the coding region of the Ogg1 gene in BTBR T+tf/J and C57BL/6J mice. (B) Bisulfite sequencing analysis of the Ogg1 promoter methylation. (C) Western blot analysis of TSC2 and AKT in the cerebellum of BTBR T+tf/J and C57BL/6J mice. (TIF) [file pone.0113712.s002.tif]
