# Supplementary material for: Cerebellar Oxidative DNA Damage and Altered DNA Methylation in the BTBR T+tf/J Mouse Model of Autism and Similarities with Human Post Mortem Cerebellum
Source: PLoS One. 2014 Nov 25;9(11):e113712. doi: 10.1371/journal.pone.0113712 (PMC4244134; doi:10.1371/journal.pone.0113712)
Supplement: Table S1 — Common differentially expressed genes in the cerebellum of BTBRT+tf/J and C57BL/6J mice. (n = 4). (DOC) [file pone.0113712.s003.doc]

**Supplementary Table 1.** Common differentially expressed genes in the cerebellum of BTBRT+tf/J and C57BL/6J mice (n = 4).

| **#** | **Gene bank**  **accession #** | **Gene name** | **Gene description** | **Male** | | **Female** | |
| --- | --- | --- | --- | --- | --- | --- | --- |
|  |  |  |  | **Fold change** | **p value** | **Fold change** | **P value** |
| 1 | NM_001037713 | Xaf1 | XIAP associated factor 1 | **238.25** | 0.0001 | **211.24** | p<0.0001 |
| 2 | NM_009999 | Cyp2b10 | Cytochrome P450, family 2, subfamily b, polypeptide 10 | **89.65** | 0.0126 | **25.36** | 0.0008 |
| 3 | AF365933 | Zfp264 | Zinc finger protein 264 | **31.02** | 0.0001 | **65.87** | 0.0001 |
| 4 | NM_023624 | Lrat | Lecithin-retinol acyltransferase (phosphatidylcholine-retinol-O-acyltransferase) | **22.82** | 0.0003 | **19.50** | 0.0003 |
| 5 | NM_010259 | Gbp1 | Guanylate binding protein 1 | **22.61** | 0.0001 | **23.19** | 0.0002 |
| 6 | NM_029210 | Sv2c | Synaptic vesicle glycoprotein 2c | **19.59** | 0.0040 | **28.89** | 0.0004 |
| 7 | NM_009246 | Serpina1d | Serine (or cysteine) peptidase inhibitor, clade A, member 1D | **16.09** | 0.0020 | **41.84** | 0.0026 |
| 8 | NM_033325 | Loxl2 | Lysyl oxidase-like 2 | **15.89** | 0.0091 | **12.02** | 0.0150 |
| 9 | NM_021475 | Adamdec1 | ADAM-like, decysin 1 | **15.83** | 0.0224 | **10.24** | 0.0008 |
| 10 | NM_177361 | Ifna12 | Interferon alpha 12 | **15.56** | 0.0003 | **15.40** | p<0.0001 |
| 11 | NM_022420 | Gprc5b | G protein-coupled receptor, family C, group 5, member B | **14.23** | 0.0001 | **6.35** | 0.0056 |
| 12 | NM_009245 | Serpina1c | Serine (or cysteine) peptidase inhibitor, clade A, member 1C | **14.23** | 0.0014 | **40.35** | 0.0049 |
| 13 | NM_026236 | Wdr48 | WD repeat domain 48 | **13.62** | p<0.0001 | **15.51** | p<0.0001 |
| 14 | NM_025951 | Pi4k2b | Phosphatidylinositol 4-kinase type 2 beta | **13.10** | 0.0101 | **7.41** | p<0.0001 |
| 15 | NM_009252 | Serpina3n | Serine (or cysteine) peptidase inhibitor, clade A, member 3N | **13.08** | 0.0106 | **13.37** | 0.0116 |
| 16 | NM_027588 | Nt5c1b | 5'-nucleotidase, cytosolic IB | **12.88** | 0.0219 | **24.00** | 0.0189 |
| 17 | NM_009243 | Serpina1a | Serine (or cysteine) peptidase inhibitor, clade A, member 1A | **12.83** | 0.0032 | **26.80** | 0.0070 |
| 18 | NM_011414 | Slpi | Secretory leukocyte peptidase inhibitor | **12.26** | 0.0026 | **11.72** | 0.0086 |
| 19 | AK090084 | Rbm45 | RNA binding motif protein 45 | **12.19** | p<0.0001 | **2.31** | 0.0002 |
| 20 | NM_027839 | Ceacam20 | Carcinoembryonic antigen-related cell adhesion molecule 20 | **11.32** | 0.0487 | **13.99** | 0.0076 |
| 21 | NM_009247 | Serpina1e | Serine (or cysteine) peptidase inhibitor, clade A, member 1E | **10.67** | 0.0019 | **14.99** | 0.0133 |
| 22 | NM_001127188 | Zfp534 | Zinc finger protein 534 | **9.75** | 0.0015 | **7.31** | 0.0045 |
| 23 | NM_009892 | Chi3l3 | Chitinase 3-like 3 | **8.86** | p<0.0001 | **17.45** | 0.0017 |
| 24 | NM_010401 | Hal | Histidine ammonia lyase | **8.84** | 0.0008 | **8.80** | 0.0027 |
| 25 | NM_146694 | Olfr1466 | Olfactory receptor 1466 | **8.71** | 0.0001 | **9.40** | 0.0009 |
| 26 | NM_177845 | Pla2g4e | Phospholipase A2, group IVE | **8.69** | 0.0005 | **8.22** | p<0.0001 |
| 27 | NM_010690 | Lats1 | Large tumor suppressor | **8.51** | 0.0017 | **9.68** | 0.0002 |
| 28 | NM_198013 | Cuedc1 | CUE domain containing 1 | **7.78** | p<0.0001 | **9.39** | p<0.0001 |
| 29 | AK046830 | Prune2 | Prune homolog 2 | **7.46** | 0.0017 | **7.36** | 0.0011 |
| 30 | NM_010767 | Masp2 | Mannan-binding lectin serine peptidase 2 | **7.38** | 0.0005 | **12.44** | 0.0001 |
| 31 | NM_172857 | Exd1 | Exonuclease 3'-5' domain containing 1 | **6.74** | 0.0002 | **4.74** | p<0.0001 |
| 32 | NM_172382 | Kdm4a | Lysine (K)-specific demethylase 4A | **6.46** | 0.0031 | **9.27** | p<0.0001 |
| 33 | NM_177337 | Arl11 | ADP-ribosylation factor-like 11 | **6.45** | 0.0071 | **6.11** | 0.0027 |
| 34 | NM_019740 | Foxo3 | Forkhead box O3 | **6.42** | 0.0006 | **5.60** | 0.0006 |
| 35 | NM_008561 | Mc3r | Melanocortin 3 receptor | **6.32** | 0.0198 | **11.05** | 0.0086 |
| 36 | NM_001037712 | Kcnh6 | Potassium voltage-gated channel, subfamily H (eag-related), member 6 | **6.23** | 0.0021 | **5.77** | 0.0051 |
| 37 | NM_177347 | Ifna13 | Interferon alpha 13 | **6.08** | 0.0246 | **5.72** | p<0.0001 |
| 38 | NM_010400 | H60a | Histocompatibility 60a | **6.06** | 0.0104 | **12.57** | 0.0003 |
| 39 | NM_010244 | Fv1 | Friend virus susceptibility 1 | **5.95** | 0.0016 | **7.76** | 0.0010 |
| 40 | NM_011940 | Ifi202b | Interferon activated gene 202B | **5.92** | 0.0143 | **5.35** | 0.0370 |
| 41 | NM_018803 | Syt10 | Synaptotagmin X | **5.81** | 0.0002 | **4.89** | 0.0006 |
| 42 | NM_009047 | Rem1 | Rad and gem related GTP binding protein 1 | **5.75** | 0.0002 | **7.98** | 0.0002 |
| 43 | NM_001113533 | Wtap | Wilms' tumour 1-associating protein | **5.65** | 0.0006 | **5.76** | 0.0002 |
| 44 | NM_178924 | Upk1b | Uroplakin 1B | **5.63** | 0.0310 | **2.50** | 0.0255 |
| 45 | NM_133997 | Apof | Apolipoprotein F | **5.61** | 0.0039 | **5.81** | 0.0160 |
| 46 | NM_133984 | Hemk1 | Hemk methyltransferase family member 1 | **5.53** | p<0.0001 | **5.14** | p<0.0001 |
| 47 | NM_009216 | Sstr1 | Somatostatin receptor 1 | **5.49** | 0.0084 | **3.76** | 0.0363 |
| 48 | NM_009036 | Rbpjl | Recombination signal binding protein for immunoglobulin kappa J region-like | **5.17** | 0.0014 | **5.66** | 0.0006 |
| 49 | BC054111 | Ap1s3 | Adaptor-related protein complex AP-1, sigma 3 | **5.13** | 0.0030 | **7.29** | 0.0008 |
| 50 | NM_007598 | Cap1 | CAP, adenylate cyclase-associated protein 1 | **4.98** | 0.0101 | **6.39** | p<0.0001 |
| 51 | NM_026812 | Hddc3 | HD domain containing 3 | **4.91** | p<0.0001 | **5.18** | p<0.0001 |
| 52 | NM_008634 | Mtap1b | Microtubule-associated protein 1B | **4.78** | 0.0007 | **4.62** | 0.0021 |
| 53 | NM_021304 | Abhd1 | Abhydrolase domain containing 1 | **4.76** | 0.0003 | **4.73** | 0.0002 |
| 54 | NM_018731 | Atp4a | Atpase, H+/K+ exchanging, gastric, alpha polypeptide | **4.74** | 0.0076 | **5.02** | 0.0058 |
| 55 | NM_172733 | Dera | 2-deoxyribose-5-phosphate aldolase homolog | **4.72** | 0.0001 | **4.81** | p<0.0001 |
| 56 | AK031929 | Socs6 | Suppressor of cytokine signaling 6 | **4.69** | 0.0028 | **3.41** | 0.0264 |
| 57 | NM_013637 | Prm1 | Protamine 1 | **4.66** | 0.0001 | **4.65** | 0.0010 |
| 58 | NM_134216 | V1rh7 | Vomeronasal 1 receptor 206 | **4.64** | 0.0008 | **3.19** | 0.0011 |
| 59 | NM_020275 | Tnfrsf10b | Tumor necrosis factor receptor superfamily, member 10b | **4.61** | p<0.0001 | **4.51** | 0.0001 |
| 60 | NM_130456 | Nphs2 | Nephrosis 2 homolog, podocin | **4.40** | 0.0055 | **3.49** | 0.0093 |
| 61 | NM_013743 | Pdk4 | Pyruvate dehydrogenase kinase, isoenzyme 4 | **4.29** | 0.0011 | **3.21** | 0.0230 |
| 62 | NM_011332 | Ccl17 | Chemokine (C-C motif) ligand 17 | **4.08** | 0.0095 | **4.20** | 0.0106 |
| 63 | NM_001111279 | Wdfy1 | WD repeat and FYVE domain containing 1 | **4.08** | 0.0007 | **3.65** | 0.0005 |
| 64 | NM_011436 | Sorl1 | Sortilin-related receptor, LDLR class A repeats-containing | **4.05** | 0.0011 | **3.10** | 0.0007 |
| 65 | NM_146559 | Olfr868 | Olfactory receptor 868 | **4.02** | 0.0337 | **4.03** | 0.0191 |
| 66 | NM_001113406 | Krtap11-1 | Keratin associated protein 11-1 | **3.93** | 0.0404 | **6.45** | 0.0177 |
| 67 | NM_009230 | Soat1 | Sterol O-acyltransferase 1 | **3.91** | 0.0005 | **3.03** | 0.0014 |
| 68 | NM_001033324 | Zbtb16 | Zinc finger and BTB domain containing 16 | **3.87** | 0.0020 | **2.67** | 0.0184 |
| 69 | NM_001005788 | Zfp69 | Zinc finger protein 69 | **3.83** | 0.0197 | **3.30** | 0.0366 |
| 70 | NM_023434 | Tox4 | TOX high mobility group box family member 4 | **3.80** | 0.0004 | **3.38** | 0.0079 |
| 71 | NM_001029842 | Slc16a6 | Solute carrier family 16 (monocarboxylic acid transporters), member 6 | **3.76** | p<0.0001 | **2.37** | 0.0004 |
| 72 | NM_011517 | Sycp3 | Synaptonemal complex protein 3 | **3.75** | 0.0263 | **2.49** | 0.0348 |
| 73 | NM_001145972 | Gpr114 | G protein-coupled receptor 114 | **3.69** | 0.0169 | **3.24** | 0.0499 |
| 74 | NM_007847 | Defa-rs2 | Defensin, alpha, related sequence 2 | **3.64** | 0.0023 | **4.55** | 0.0031 |
| 75 | NM_007669 | Cdkn1a | Cyclin-dependent kinase inhibitor 1A (P21) | **3.58** | 0.0027 | **2.43** | 0.0084 |
| 76 | NM_027651 | Tmem30c | Transmembrane protein 30C | **3.58** | 0.0128 | **3.28** | 0.0225 |
| 77 | NM_029415 | Slc10a6 | Solute carrier family 10 (sodium/bile acid cotransporter family), member 6 | **3.53** | 0.0101 | **3.04** | 0.0063 |
| 78 | NM_001081072 | Slc27a6 | Solute carrier family 27 (fatty acid transporter), member 6 | **3.41** | 0.0470 | **3.42** | 0.0053 |
| 79 | NM_011440 | Sox14 | SRY-box containing gene 14 | **3.40** | 0.0068 | **2.74** | 0.0232 |
| 80 | NM_001033415 | Shisa3 | Shisa homolog 3 (Xenopus laevis) | **3.39** | 0.0411 | **4.19** | 0.0043 |
| 81 | AK164147 | Def8 | Differentially expressed in FDCP 8 | **3.39** | 0.0123 | **4.19** | 0.0019 |
| 82 | NM_174993 | Fmr1nb | Fragile X mental retardation 1 neighbor | **3.39** | 0.0129 | **2.42** | 0.0158 |
| 83 | NM_001085383 | Anxa9 | Annexin A9 | **3.35** | 0.0022 | **3.77** | 0.0004 |
| 84 | NM_177811 | Zfp459 | Zinc finger protein 459 | **3.35** | 0.0006 | **3.73** | 0.0008 |
| 85 | NM_133187 | Fam198b | Family with sequence similarity 198, member B | **3.32** | 0.0029 | **5.67** | 0.0104 |
| 86 | NM_011832 | Insrr | Insulin receptor-related receptor | **3.13** | 0.0354 | **2.76** | 0.0173 |
| 87 | NM_018777 | Cldn6 | Claudin 6 | **3.07** | 0.0023 | **2.13** | 0.0139 |
| 88 | NM_025394 | Tomm7 | Translocase of outer mitochondrial membrane 7 homolog | **3.03** | 0.0002 | **2.99** | 0.0001 |
| 89 | NM_020272 | Pik3cg | Phosphoinositide-3-kinase, catalytic, gamma polypeptide | **2.99** | 0.0226 | **3.21** | 0.0374 |
| 90 | NM_026473 | Tubb6 | Tubulin, beta 6 | **2.97** | 0.0002 | **2.64** | 0.0043 |
| 91 | NM_145395 | Duoxa1 | Dual oxidase maturation factor 1 | **2.96** | 0.0263 | **2.68** | 0.0418 |
| 92 | NM_029926 | Irak4 | Interleukin-1 receptor-associated kinase 4 | **2.95** | 0.0372 | **2.33** | 0.0418 |
| 93 | NM_016868 | Hif3a | Hypoxia inducible factor 3, alpha subunit | **2.94** | 0.0071 | **5.62** | 0.0038 |
| 94 | NM_053247 | Lyve1 | Lymphatic vessel endothelial hyaluronan receptor 1 | **2.92** | 0.0156 | **2.60** | 0.0404 |
| 95 | NM_008710 | Nnt | Nicotinamide nucleotide transhydrogenase | **2.88** | 0.0249 | **3.24** | 0.0063 |
| 96 | NM_001081299 | Cdh18 | Cadherin 18 | **2.85** | 0.0230 | **2.63** | 0.0109 |
| 97 | NM_177366 | Gpr157 | G protein-coupled receptor 157 | **2.85** | 0.0026 | **3.28** | 0.0131 |
| 98 | BC016565 | Ppih | Peptidyl prolyl isomerase H | **2.83** | 0.0164 | **3.98** | 0.0003 |
| 99 | NM_011095 | Lilrb3 | Leukocyte immunoglobulin-like receptor, subfamily B (with TM and ITIM domains), member 3 | **2.79** | 0.0345 | **5.05** | 0.0049 |
| 100 | NM_025378 | Ifitm3 | Interferon induced transmembrane protein 3 | **2.77** | 0.0010 | **2.52** | 0.0157 |
| 101 | NM_019658 | Shoc2 | Soc-2 (suppressor of clear) homolog | **2.75** | 0.0001 | **2.64** | p<0.0001 |
| 102 | NM_182782 | Klhl25 | Kelch-like 25 | **2.74** | 0.0398 | **3.20** | 0.0039 |
| 103 | NM_146106 | Lyplal1 | Lysophospholipase-like 1 | **2.73** | 0.0016 | **2.50** | 0.0044 |
| 104 | NM_007843 | Defb1 | Defensin beta 1 | **2.70** | 0.0055 | **3.49** | 0.0060 |
| 105 | NM_001145060 | Glyatl3 | Glycine-N-acyltransferase-like 3 | **2.69** | 0.0041 | **3.35** | 0.0044 |
| 106 | NM_001163457 | Mttp | Microsomal triglyceride transfer protein | **2.65** | 0.0006 | **2.74** | 0.0028 |
| 107 | M60419 | Ybx1 | Y box protein 1 | **2.63** | p<0.0001 | **2.76** | 0.0002 |
| 108 | NM_001101464 | Foxi3 | Forkhead box I3 | **2.62** | 0.0331 | **2.38** | 0.0387 |
| 109 | NM_146902 | Olfr1221 | Olfactory receptor 1221 | **2.61** | 0.0169 | **3.12** | 0.0051 |
| 110 | NM_027495 | Tmem144 | Transmembrane protein 144 | **2.60** | 0.0060 | **2.19** | 0.0092 |
| 111 | NM_027389 | Gon4l | Gon-4-like (C.elegans) | **2.58** | 0.0065 | **2.24** | 0.0027 |
| 112 | NM_134052 | Adi1 | Acireductone dioxygenase 1 | **2.58** | 0.0003 | **2.49** | 0.0030 |
| 113 | AK138305 | Trp63 | Transformation related protein 63 | **2.58** | 0.0040 | **4.60** | 0.0023 |
| 114 | NM_016785 | Tpmt | Thiopurine methyltransferase | **2.57** | 0.0003 | **2.10** | 0.0011 |
| 115 | NM_016770 | Folh1 | Folate hydrolase | **2.54** | 0.0150 | **2.31** | 0.0212 |
| 116 | NM_011933 | Decr2 | 2-4-dienoyl-Coenzyme A reductase 2, peroxisomal | **2.51** | 0.0042 | **2.04** | 0.0015 |
| 117 | NM_011067 | Per3 | Period homolog 3 | **2.50** | 0.0176 | **4.94** | 0.0016 |
| 118 | NM_001114347 | Clasp2 | CLIP associating protein 2 | **2.50** | 0.0003 | **2.88** | 0.0016 |
| 119 | NM_026840 | Pdgfrl | Platelet-derived growth factor receptor-like | **2.49** | 0.0002 | **2.42** | 0.0002 |
| 120 | NM_001080969 | Thg1l | Trna-histidine guanylyltransferase 1-like | **2.48** | 0.0123 | **2.37** | 0.0006 |
| 121 | NM_176972 | Usp37 | Ubiquitin specific peptidase 37 | **2.47** | 0.0057 | **2.26** | 0.0008 |
| 122 | NM_178364 | Zfp369 | Zinc finger protein 369 | **2.43** | 0.0001 | **2.70** | 0.0006 |
| 123 | NM_008066 | Gabra2 | Gamma-aminobutyric acid (GABA) A receptor, subunit alpha 2 | **2.42** | 0.0282 | **2.78** | 0.0027 |
| 124 | NM_027741 | Mro | Maestro | **2.38** | 0.0117 | **2.20** | 0.0017 |
| 125 | NM_053077 | Slc45a2 | Solute carrier family 45, member 2 | **2.35** | 0.0066 | **2.33** | 0.0223 |
| 126 | NM_001110497 | Tmem87a | Transmembrane protein 87A | **2.35** | 0.0003 | **2.82** | 0.0025 |
| 127 | NM_145572 | Gys2 | Glycogen synthase 2 | **2.35** | 0.0125 | **3.05** | 0.0461 |
| 128 | NM_010180 | Fbln1 | Fibulin 1 | **2.33** | 0.0173 | **3.13** | 0.0205 |
| 129 | NM_001081418 | Gltscr1 | Glioma tumor suppressor candidate region gene 1 | **2.32** | 0.0124 | **2.59** | 0.0103 |
| 130 | NM_013634 | Med1 | Mediator complex subunit 1 | **2.31** | p<0.0001 | **2.13** | 0.0031 |
| 131 | NM_206975 | Ifna14 | Interferon, alpha 14 | **2.29** | 0.0208 | **2.63** | 0.0057 |
| 132 | NM_009162 | Scg5 | Secretogranin V | **2.27** | 0.0001 | **2.44** | p<0.0001 |
| 133 | NM_001077410 | Gimap8 | Gtpase, IMAP family member 8 | **2.19** | p<0.0001 | **2.11** | 0.0132 |
| 134 | NM_177733 | E2f2 | E2F transcription factor 2 | **2.17** | 0.0383 | **2.56** | 0.0221 |
| 135 | NM_008629 | Msi1 | Musashi homolog 1(Drosophila) | **2.16** | 0.0079 | **2.22** | 0.0357 |
| 136 | NM_172878 | Ttc34 | Tetratricopeptide repeat domain 34 | **2.15** | 0.0202 | **2.99** | 0.0008 |
| 137 | NM_008813 | Enpp1 | Ectonucleotide pyrophosphatase/phosphodiesterase 1 | **2.11** | 0.0150 | **2.53** | 0.0162 |
| 138 | NM_001104539 | Vmn2r90 | Vomeronasal 2, receptor 90 | **2.07** | 0.0227 | **4.93** | 0.0119 |
| 139 | NM_027644 | Prss41 | Protease, serine, 41 | **2.07** | 0.0036 | **2.31** | 0.0006 |
| 140 | NM_008336 | Ifnab | Interferon alpha B | **2.07** | 0.0072 | **2.32** | 0.0008 |
| 141 | NM_028622 | Lce1c | Late cornified envelope 1C | **2.06** | 0.0236 | **2.60** | 0.0378 |
| 142 | NM_145505 | Fam160b1 | Family with sequence similarity 160, member B1 | **2.05** | p<0.0001 | **2.23** | 0.0001 |
| 143 | NM_175229 | Srrm2 | Serine/arginine repetitive matrix 2 | **2.03** | 0.0070 | **2.21** | 0.0031 |
| 144 | NM_146155 | Ahdc1 | AT hook, DNA binding motif, containing 1 | **2.02** | 0.0003 | **2.01** | 0.0024 |
| 145 | NR_015348 | Hoxa11as | HOXA11 antisense RNA (non-protein coding) | **-2.02** | 0.0029 | **-2.51** | 0.0004 |
| 146 | NM_001005506 | Txlna | Taxilin alpha | **-2.02** | 0.0017 | **-2.56** | 0.0048 |
| 147 | NM_133925 | Rbm28 | RNA binding motif protein 28 | **-2.02** | 0.0001 | **-2.20** | p<0.0001 |
| 148 | NM_177323 | Rint1 | RAD50 interactor 1 | **-2.02** | 0.0086 | **-2.13** | 0.0207 |
| 149 | NM_023058 | Pkmyt1 | Protein kinase, membrane associated tyrosine/threonine 1 | **-2.03** | p<0.0001 | **-2.11** | 0.0007 |
| 150 | NM_025575 | Sys1 | SYS1 Golgi-localized integral membrane protein homolog | **-2.05** | 0.0001 | **-2.42** | 0.0001 |
| 151 | NM_028876 | Tmed5 | Transmembrane emp24 protein transport domain containing 5 | **-2.05** | 0.0001 | **-2.05** | 0.0004 |
| 152 | NM_001164237 | Rnf41 | Ring finger protein 41 | **-2.08** | 0.0034 | **-2.38** | 0.0032 |
| 153 | NM_008250 | Hlx | H2.0-like homeobox | **-2.10** | 0.0123 | **-2.53** | 0.0154 |
| 154 | NM_172919 | Zfp846 | Zinc finger protein 846 | **-2.10** | 0.0086 | **-2.52** | 0.0032 |
| 155 | NM_007631 | Ccnd1 | Cyclin D1 | **-2.10** | 0.0005 | **-2.33** | 0.0001 |
| 156 | NM_178698 | Pigv | Phosphatidylinositol glycan anchor biosynthesis, class V | **-2.10** | p<0.0001 | **-2.71** | 0.0024 |
| 157 | NM_027123 | Fastkd3 | FAST kinase domains 3 | **-2.12** | 0.0109 | **-2.44** | 0.0058 |
| 158 | NM_001080967 | Tmsb15b2 | Thymosin beta 15b2 | **-2.13** | 0.0002 | **-2.03** | 0.0003 |
| 159 | NM_011500 | Strn | Striatin, calmodulin binding protein | **-2.14** | 0.0225 | **-2.16** | 0.0027 |
| 160 | NM_023284 | Nuf2 | NUF2, NDC80 kinetochore complex component, homolog | **-2.15** | 0.0199 | **-2.99** | 0.0134 |
| 161 | NM_025961 | Gatm | Glycine amidinotransferase (L-arginine:glycine amidinotransferase) | **-2.16** | 0.0388 | **-2.86** | 0.0219 |
| 162 | NM_028243 | Prcp | Prolylcarboxypeptidase (angiotensinase C) | **-2.17** | 0.0385 | **-4.33** | 0.0039 |
| 163 | NM_001168392 | Ramp1 | Receptor (calcitonin) activity modifying protein 1 | **-2.17** | 0.0076 | **-2.31** | 0.0192 |
| 164 | NM_026855 | Arv1 | ARV1 homolog | **-2.19** | 0.0280 | **-2.06** | 0.0408 |
| 165 | NM_178194 | Hist1h2be | Histone cluster 1, h2be | **-2.19** | 0.0464 | **-2.39** | 0.0056 |
| 166 | NM_053169 | Trim16 | Tripartite motif-containing 16 | **-2.21** | 0.0217 | **-4.28** | 0.0051 |
| 167 | NM_152814 | Zfp566 | Zinc finger protein 566 | **-2.23** | 0.0001 | **-2.07** | 0.0001 |
| 168 | NM_178208 | Hist1h4c | Histone cluster 1, h4c | **-2.23** | 0.0003 | **-2.12** | 0.0119 |
| 169 | NM_001110239 | Acp1 | Acid phosphatase 1, soluble | **-2.23** | 0.0013 | **-2.41** | 0.0002 |
| 170 | NM_001012434 | Kctd14 | Potassium channel tetramerisation domain containing 14 | **-2.24** | 0.0232 | **-3.61** | 0.0039 |
| 171 | NM_133659 | Erg | Avian erythroblastosis virus E-26 (v-ets) oncogene related | **-2.26** | 0.0347 | **-2.46** | 0.0177 |
| 172 | NM_172529 | Gnptg | N-acetylglucosamine-1-phosphotransferase, gamma subunit | **-2.29** | 0.0125 | **-3.11** | 0.0004 |
| 173 | NM_028095 | Mettl10 | Methyltransferase like 10 | **-2.30** | 0.0010 | **-2.03** | 0.0087 |
| 174 | NM_008228 | Hdac1 | Histone deacetylase 1 | **-2.31** | 0.0022 | **-2.17** | 0.0041 |
| 175 | NM_001033300 | Gmps | Guanine monophosphate synthetase | **-2.32** | 0.0008 | **-2.15** | 0.0050 |
| 176 | NM_001136073 | Nfatc2 | Nuclear factor of activated T-cells, cytoplasmic, calcineurin-dependent 2 | **-2.35** | 0.0099 | **-2.56** | 0.0039 |
| 177 | NM_027947 | Zbtb43 | Zinc finger and BTB domain containing 43 | **-2.36** | 0.0012 | **-2.30** | 0.0058 |
| 178 | NM_026817 | Rabl2a | RAB, member of RAS oncogene family-like 2 | **-2.36** | 0.0002 | **-2.68** | 0.0003 |
| 179 | NM_026793 | Myct1 | Myc target 1 | **-2.37** | 0.0361 | **-2.70** | 0.0013 |
| 180 | NM_178684 | Mapk1ip1l | Mitogen-activated protein kinase 1 interacting protein 1-like | **-2.40** | 0.0049 | **-2.09** | 0.0064 |
| 181 | NM_001033960 | Rabgap1 | RAB gtpase activating protein 1 | **-2.42** | 0.0013 | **-2.04** | 0.0191 |
| 182 | NM_020557 | Cmpk2 | Cytidine monophosphate (UMP-CMP) kinase 2, mitochondrial | **-2.46** | 0.0164 | **-3.34** | 0.0049 |
| 183 | NM_080708 | Bmp2k | BMP2 inducible kinase | **-2.46** | 0.0253 | **-2.55** | 0.0103 |
| 184 | NM_025314 | Dtd1 | D-tyrosyl-trna deacylase 1 homolog | **-2.47** | 0.0003 | **-2.32** | 0.0010 |
| 185 | NM_025898 | Napa | N-ethylmaleimide sensitive fusion protein attachment protein alpha | **-2.49** | 0.0053 | **-2.05** | 0.0219 |
| 186 | NM_011499 | Strap | Serine/threonine kinase receptor associated protein | **-2.50** | 0.0005 | **-2.18** | 0.0001 |
| 187 | NM_145619 | Parp3 | Poly (ADP-ribose) polymerase family, member 3 | **-2.50** | p<0.0001 | **-2.54** | p<0.0001 |
| 188 | NM_026858 | Xrcc6bp1 | XRCC6 binding protein 1 | **-2.50** | 0.0084 | **-2.80** | 0.0038 |
| 189 | NM_026082 | Dock7 | Dedicator of cytokinesis 7 | **-2.51** | 0.0010 | **-2.64** | p<0.0001 |
| 190 | NM_027921 | Slc16a14 | Solute carrier family 16 (monocarboxylic acid transporters), member 14 | **-2.53** | 0.0272 | **-2.29** | 0.0264 |
| 191 | NR_028331 | Zfp672 | Zinc finger protein 672 | **-2.53** | 0.0033 | **-2.38** | 0.0146 |
| 192 | NM_019552 | Abcb10 | ATP-binding cassette, sub-family B (MDR/TAP), member 10 | **-2.56** | 0.0054 | **-2.31** | 0.0012 |
| 193 | AK049583 | Mfn2 | Mitofusin 2 | **-2.56** | 0.0099 | **-2.88** | 0.0439 |
| 194 | NM_001013362 | Cbx6-Nptxr | Neuronal pentraxin chromo domain | **-2.57** | 0.0212 | **-2.25** | 0.0071 |
| 195 | NM_028121 | Adpgk | ADP-dependent glucokinase | **-2.57** | 0.0183 | **-3.21** | 0.0115 |
| 196 | NM_026211 | Tmed9 | Transmembrane emp24 protein transport domain containing 9 | **-2.60** | p<0.0001 | **-2.54** | p<0.0001 |
| 197 | NM_001005605 | Aebp2 | AE binding protein 2 | **-2.60** | 0.0011 | **-2.43** | 0.0075 |
| 198 | NM_175154 | Galk2 | Galactokinase 2 | **-2.61** | 0.0016 | **-2.34** | 0.0029 |
| 199 | NM_001083318 | Etv3 | Ets variant gene 3 | **-2.63** | p<0.0001 | **-3.21** | p<0.0001 |
| 200 | NM_025298 | Polr3e | Polymerase (RNA) III (DNA directed) polypeptide E | **-2.63** | 0.0004 | **-2.41** | p<0.0001 |
| 201 | NM_010865 | Myoc | Myocilin | **-2.65** | 0.0072 | **-3.81** | 0.0085 |
| 202 | NM_028838 | Lrrc2 | Leucine rich repeat containing 2 | **-2.66** | 0.0444 | **-2.42** | 0.0396 |
| 203 | NM_053196 | Sfxn2 | Sideroflexin 2 | **-2.67** | 0.0037 | **-2.14** | 0.0076 |
| 204 | NM_013659 | Sema4b | Sema domain, immunoglobulin domain (Ig), transmembrane domain (TM) and short cytoplasmic domain, (semaphorin) 4B | **-2.68** | 0.0007 | **-2.02** | 0.0002 |
| 205 | NM_001167883 | Ankrd50 | Ankyrin repeat domain 50 | **-2.69** | 0.0005 | **-2.10** | 0.0090 |
| 206 | NM_178892 | Tiparp | TCDD-inducible poly(ADP-ribose) polymerase | **-2.70** | p<0.0001 | **-2.94** | 0.0001 |
| 207 | NM_001081983 | Tmsb15b1 | Thymosin beta 15b1 | **-2.70** | p<0.0001 | **-2.76** | 0.0005 |
| 208 | NM_023493 | Cml5 | Camello-like 5 | **-2.70** | 0.0004 | **-5.30** | p<0.0001 |
| 209 | NM_199062 | Zfp781 | Zinc finger protein 781 | **-2.73** | 0.0020 | **-2.44** | 0.0219 |
| 210 | NM_016780 | Itgb3 | Integrin beta 3 | **-2.73** | 0.0277 | **-3.74** | 0.0191 |
| 211 | NM_008064 | Gaa | Glucosidase, alpha, acid | **-2.74** | 0.0413 | **-2.45** | 0.0211 |
| 212 | NM_013603 | Mt3 | Metallothionein 3 | **-2.76** | 0.0008 | **-2.75** | 0.0009 |
| 213 | NM_001081264 | Alg6 | Asparagine-linked glycosylation 6 homolog (yeast, alpha-1,3,-glucosyltransferase) | **-2.76** | 0.0018 | **-2.34** | 0.0057 |
| 214 | NM_019440 | Irgm2 | Immunity-related gtpase family M member 2 | **-2.77** | 0.0056 | **-2.79** | 0.0178 |
| 215 | NM_027354 | Wdr51a | WD repeat domain 51 alpha | **-2.79** | 0.0498 | **-3.08** | 0.0046 |
| 216 | XM_205565 | Rsph10b2 | Radial spoke head 10 homolog B | **-2.81** | 0.0043 | **-2.39** | 0.0074 |
| 217 | NM_016846 | Rgl1 | Ral guanine nucleotide dissociation stimulator,-like 1 | **-2.84** | 0.0207 | **-2.90** | 0.0173 |
| 218 | NM_172921 | Fam55d | Family with sequence similarity 55, member D | **-2.86** | 0.0064 | **-2.03** | 0.0041 |
| 219 | NM_177688 | H2afj | H2A histone family, member J | **-2.87** | p<0.0001 | **-2.85** | 0.0027 |
| 220 | BC019757 | Hist1h4i | Histone cluster 1, h4i | **-2.91** | 0.0005 | **-3.16** | 0.0005 |
| 221 | NM_018867 | Cpxm2 | Carboxypeptidase X 2 (M14 family) | **-2.92** | 0.0107 | **-3.05** | 0.0371 |
| 222 | NM_198652 | Hjurp | Holliday junction recognition protein | **-2.97** | 0.0178 | **-4.65** | 0.0013 |
| 223 | NM_001081642 | Xlr4a | X-linked lymphocyte-regulated 4A | **-2.99** | 0.0082 | **-2.41** | 0.0304 |
| 224 | NM_026218 | Fgfr1op2 | FGFR1 oncogene partner 2 | **-3.05** | 0.0019 | **-3.55** | 0.0054 |
| 225 | NM_001039038 | Nhlrc4 | NHL repeat containing 4 | **-3.08** | 0.0280 | **-2.17** | 0.0098 |
| 226 | NM_175000 | Hbq1 | Hemoglobin, theta 1A | **-3.10** | 0.0178 | **-3.11** | 0.0081 |
| 227 | NM_022563 | Ddr2 | Discoidin domain receptor family, member 2 | **-3.11** | 0.0021 | **-3.36** | 0.0021 |
| 228 | NM_009096 | Rps6 | Ribosomal protein S6 | **-3.18** | 0.0027 | **-3.09** | 0.0003 |
| 229 | NM_019940 | Zfp111 | Zinc finger protein 111 | **-3.21** | 0.0041 | **-2.50** | 0.0229 |
| 230 | NM_029631 | Abhd14b | Abhydrolase domain containing 14b | **-3.22** | 0.0053 | **-2.84** | 0.0283 |
| 231 | NM_177832 | Zfp236 | Zinc finger protein 236 | **-3.24** | 0.0197 | **-2.48** | 0.0035 |
| 232 | NM_011980 | Zfp146 | Zinc finger protein 146 | **-3.26** | p<0.0001 | **-3.36** | 0.0015 |
| 233 | NM_025915 | Tmem88 | Transmembrane protein 88 | **-3.28** | 0.0004 | **-2.74** | 0.0007 |
| 234 | NM_175113 | Trmt6 | Trna methyltransferase 6 homolog | **-3.30** | 0.0108 | **-2.88** | 0.0012 |
| 235 | NM_026725 | Dusp23 | Dual specificity phosphatase 23 | **-3.35** | 0.0009 | **-3.36** | p<0.0001 |
| 236 | NM_177867 | Spata21 | Spermatogenesis associated 21 | **-3.40** | 0.0002 | **-2.47** | 0.0019 |
| 237 | NM_008357 | Il15 | Interleukin 15 | **-3.42** | 0.0065 | **-2.96** | 0.0380 |
| 238 | NM_011819 | Gdf15 | Growth differentiation factor 15 | **-3.44** | 0.0200 | **-5.01** | 0.0103 |
| 239 | NM_001105152 | Vmn2r48 | Vomeronasal 2, receptor 48 | **-3.48** | 0.0028 | **-3.63** | 0.0032 |
| 240 | NM_010957 | Ogg1 | 8-oxoguanine DNA-glycosylase 1 | **-3.50** | 0.0010 | **-3.53** | 0.0007 |
| 241 | NM_001037758 | Btrc | Beta-transducin repeat containing protein | **-3.51** | 0.0008 | **-3.45** | 0.0086 |
| 242 | AK079707 | Manbal | Mannosidase, beta A, lysosomal-like | **-3.51** | 0.0380 | **-3.90** | 0.0030 |
| 243 | NM_033620 | Pard3 | Par-3 (partitioning defective 3) homolog | **-3.56** | 0.0061 | **-4.62** | 0.0010 |
| 244 | NM_177378 | Rnf150 | Ring finger protein 150 | **-3.57** | 0.0270 | **-2.07** | 0.0036 |
| 245 | NM_177074 | Slc38a11 | Solute carrier family 38, member 11 | **-3.57** | 0.0004 | **-4.99** | 0.0025 |
| 246 | NM_030026 | Mccc2 | Methylcrotonoyl-Coenzyme A carboxylase 2 (beta) | **-3.59** | p<0.0001 | **-3.32** | 0.0027 |
| 247 | NM_001045481 | Ifi203 | Interferon activated gene 203 | **-3.63** | 0.0179 | **-4.89** | 0.0022 |
| 248 | NM_178183 | Hist1h2ak | Histone cluster 1, h2ak | **-3.64** | 0.0140 | **-3.43** | p<0.0001 |
| 249 | NM_207229 | Plac9 | Placenta specific 9 | **-3.68** | 0.0058 | **-3.08** | 0.0185 |
| 250 | NM_153421 | Phc3 | Polyhomeotic-like 3 | **-3.70** | 0.0024 | **-3.55** | 0.0066 |
| 251 | NM_176912 | Gpr77 | G protein-coupled receptor 77 | **-3.74** | 0.0027 | **-3.83** | 0.0015 |
| 252 | NM_178210 | Hist1h4j | Histone cluster 1, h4j | **-3.79** | p<0.0001 | **-4.42** | 0.0005 |
| 253 | NM_178788 | Dctd | Dcmp deaminase | **-3.83** | 0.0054 | **-3.73** | 0.0207 |
| 254 | NM_001113481 | Sema4f | Sema domain, immunoglobulin domain (Ig), TM domain, and short cytoplasmic domain | **-3.89** | 0.0280 | **-5.00** | 0.0042 |
| 255 | NM_144527 | Ccdc21 | Coiled-coil domain containing 21 | **-3.91** | 0.0001 | **-3.46** | p<0.0001 |
| 256 | NM_001105065 | Vmn2r33 | Vomeronasal 2, receptor33 | **-4.00** | 0.0426 | **-4.00** | 0.0088 |
| 257 | NM_001114637 | Jmjd7 | Jumonji domain containing 7 | **-4.04** | 0.0050 | **-3.81** | p<0.0001 |
| 258 | NM_172461 | Nek11 | NIMA (never in mitosis gene a)-related expressed kinase 11 | **-4.06** | 0.0038 | **-6.05** | 0.0087 |
| 259 | NM_023879 | Rpgrip1 | Retinitis pigmentosa gtpase regulator interacting protein 1 | **-4.11** | 0.0023 | **-4.47** | 0.0001 |
| 260 | NM_001105076 | Vmn2r46 | Vomeronasal 2, receptor 46 | **-4.15** | 0.0060 | **-3.45** | 0.0007 |
| 261 | NM_008663 | Myo7a | Myosin VIIA | **-4.22** | 0.0018 | **-7.40** | p<0.0001 |
| 262 | NM_001033450 | Mnda | Myeloid cell nuclear differentiation antigen | **-4.23** | 0.0241 | **-6.05** | 0.0013 |
| 263 | NM_021537 | Stk25 | Serine/threonine kinase 25 | **-4.26** | p<0.0001 | **-4.18** | p<0.0001 |
| 264 | NR_028429 | Thap6 | THAP domain containing 6 | **-4.26** | 0.0004 | **-5.20** | 0.0006 |
| 265 | NM_030684 | Trim34 | Tripartite motif-containing 34A | **-4.31** | 0.0002 | **-4.26** | 0.0045 |
| 266 | NM_175029 | Atg4c | Autophagy-related 4C | **-4.42** | 0.0054 | **-3.39** | 0.0016 |
| 267 | NM_025718 | Dnase1l2 | Deoxyribonuclease 1-like 2 | **-4.42** | 0.0039 | **-4.85** | 0.0043 |
| 268 | NM_033080 | Nudt19 | Nudix (nucleoside diphosphate linked moiety X)-type motif 19 | **-4.52** | 0.0001 | **-4.35** | 0.0001 |
| 269 | NM_134241 | V1rh18 | Vomeronasal 1 receptor 212 | **-4.54** | 0.0019 | **-3.82** | 0.0022 |
| 270 | NM_028460 | Pear1 | Platelet endothelial aggregation receptor 1 | **-4.60** | 0.0020 | **-4.48** | 0.0040 |
| 271 | NM_009341 | Tcp10c | T-complex protein 10c | **-4.66** | 0.0004 | **-2.68** | 0.0004 |
| 272 | NM_026526 | N6amt2 | N-6 adenine-specific DNA methyltransferase 2 (putative) | **-4.68** | p<0.0001 | **-4.49** | 0.0002 |
| 273 | NM_175660 | Hist1h2ab | Histone cluster 1, h2ab | **-4.77** | 0.0308 | **-3.97** | 0.0242 |
| 274 | NM_146079 | Guca1b | Guanylate cyclase activator 1B | **-4.97** | 0.0042 | **-2.37** | 0.0236 |
| 275 | NM_001145960 | Slc37a2 | Solute carrier family 37 (glycerol-3-phosphate transporter), member 2 | **-5.01** | 0.0075 | **-8.06** | 0.0043 |
| 276 | NM_175031 | Stk36 | Serine/threonine kinase 36 | **-5.04** | 0.0029 | **-6.05** | 0.0014 |
| 277 | NM_033596 | Hist2h4 | Histone cluster 2, H4 | **-5.05** | 0.0004 | **-4.91** | 0.0012 |
| 278 | NM_019393 | Exosc9 | Exosome component 9 | **-5.09** | 0.0001 | **-4.96** | 0.0036 |
| 279 | NM_175652 | Hist4h4 | Histone cluster 4, H4 | **-5.15** | p<0.0001 | **-4.87** | 0.0046 |
| 280 | NM_021467 | Tnni1 | Troponin I, skeletal, slow 1 | **-5.24** | 0.0046 | **-5.01** | 0.0278 |
| 281 | AK136394 | Zfp874 | Zinc finger protein 874 | **-5.27** | 0.0052 | **-93.46** | 0.0008 |
| 282 | AK049278 | Zfp74 | Zinc finger protein 74 | **-5.29** | 0.0083 | **-7.60** | 0.0001 |
| 283 | NM_009943 | Cox6a2 | Cytochrome c oxidase, subunit VI a, polypeptide 2 | **-5.39** | 0.0377 | **-7.52** | 0.0233 |
| 284 | NM_011562 | Tdgf1 | Teratocarcinoma-derived growth factor 1 | **-5.40** | 0.0022 | **-6.18** | 0.0008 |
| 285 | NM_178211 | Hist1h4k | Histone cluster 1, h4k | **-5.51** | p<0.0001 | **-5.38** | 0.0014 |
| 286 | NM_001013779 | Aim2 | Absent in melanoma 2 | **-5.59** | 0.0004 | **-4.31** | 0.0009 |
| 287 | NM_207666 | Dlk2 | Delta-like 2 homolog | **-5.61** | p<0.0001 | **-4.85** | 0.0142 |
| 288 | NM_007946 | Epx | Eosinophil peroxidase | **-5.64** | 0.0046 | **-11.20** | 0.0067 |
| 289 | NM_025390 | Pop4 | Processing of precursor 4, ribonuclease P/MRP family, | **-5.77** | 0.0002 | **-5.06** | p<0.0001 |
| 290 | NM_001103199 | Skint6 | Selection and upkeep of intraepithelial T cells 6 | **-5.87** | 0.0076 | **-10.83** | 0.0002 |
| 291 | NM_181545 | Slfn8 | Schlafen 8 | **-6.04** | 0.0002 | **-4.41** | 0.0018 |
| 292 | NM_007582 | Cacng1 | Calcium channel, voltage-dependent, gamma subunit 1 | **-6.07** | 0.0100 | **-2.53** | 0.0099 |
| 293 | NM_016972 | Slc7a8 | Solute carrier family 7 (cationic amino acid transporter, y+ system), member 8 | **-6.30** | 0.0002 | **-4.63** | 0.0037 |
| 294 | NM_011860 | Nlrp5 | NLR family, pyrin domain containing 5 | **-6.69** | 0.0019 | **-6.62** | 0.0035 |
| 295 | NR_003555 | Vmn2r29 | Vomeronasal 2, receptor 29 | **-6.93** | 0.0043 | **-3.60** | 0.0036 |
| 296 | NM_146949 | Olfr339 | Olfactory receptor 339 | **-7.14** | 0.0013 | **-5.95** | 0.0046 |
| 297 | NM_020002 | Rec8 | REC8 homolog | **-7.18** | 0.0056 | **-6.49** | 0.0009 |
| 298 | NM_001105067 | Vmn2r35 | Vomeronasal 2, receptor 35 | **-7.21** | 0.0015 | **-8.30** | 0.0009 |
| 299 | NM_009773 | Bub1b | Budding uninhibited by benzimidazoles 1 homolog, beta | **-7.92** | 0.0068 | **-5.52** | 0.0173 |
| 300 | NM_178020 | Hyal3 | Hyaluronoglucosaminidase 3 | **-7.99** | 0.0018 | **-6.03** | 0.0043 |
| 301 | NM_175397 | Sp110 | Sp110 nuclear body protein | **-8.09** | 0.0001 | **-3.33** | 0.0093 |
| 302 | NM_145594 | Fgl1 | Fibrinogen-like protein 1 | **-8.13** | 0.0093 | **-24.94** | 0.0008 |
| 303 | NM_178749 | Stk32a | Serine/threonine kinase 32A | **-8.17** | 0.0029 | **-13.33** | p<0.0001 |
| 304 | NM_001083342 | Ptchd2 | Patched domain containing 2 | **-8.50** | 0.0002 | **-6.09** | p<0.0001 |
| 305 | NM_008922 | Prim2 | DNA primase, p58 subunit | **-9.19** | 0.0001 | **-9.41** | p<0.0001 |
| 306 | NM_025974 | Rpl14 | Ribosomal protein L14 | **-9.67** | 0.0311 | **-4.35** | 0.0004 |
| 307 | NM_026331 | Slc25a37 | Solute carrier family 25, member 37 | **-11.70** | p<0.0001 | **-9.06** | 0.0007 |
| 308 | NM_026146 | Eps8l1 | EPS8-like 1 | **-12.35** | 0.0025 | **-7.13** | 0.0004 |
| 309 | NM_028130 | Zfp157 | Zinc finger protein 157 | **-12.85** | p<0.0001 | **-11.90** | 0.0010 |
| 310 | NM_175654 | Hist1h4d | Histone cluster 1, h4d | **-14.47** | p<0.0001 | **-14.18** | 0.0014 |
| 311 | NM_026668 | Lrriq4 | Leucine-rich repeats and IQ motif containing 4 | **-14.62** | 0.0020 | **-9.17** | 0.0100 |
| 312 | NM_011029 | Rpsa | Ribosomal protein SA | **-15.31** | 0.0001 | **-18.73** | 0.0001 |
| 313 | NM_175418 | Mybpc1 | Myosin binding protein C, slow-type | **-15.67** | 0.0374 | **-15.48** | 0.0175 |
| 314 | NM_026849 | Mtmr14 | Myotubularin related protein 14 | **-16.69** | p<0.0001 | **-14.27** | p<0.0001 |
| 315 | NM_175491 | Smcr8 | Smith-Magenis syndrome chromosome region, candidate 8 homolog | **-16.84** | p<0.0001 | **-9.99** | 0.0217 |
| 316 | NM_026153 | Ankrd33b | Ankyrin repeat domain 33B | **-20.49** | 0.0005 | **-11.49** | 0.0016 |
| 317 | NM_020279 | Ccl28 | Chemokine (C-C motif) ligand 28 | **-20.92** | 0.0004 | **-12.35** | 0.0001 |
| 318 | NM_023835 | Trim12 | Tripartite motif-containing 12A | **-22.00** | 0.0006 | **-26.14** | 0.0002 |
| 319 | NM_013917 | Pttg1 | Pituitary tumor-transforming gene 1 | **-36.30** | 0.0004 | **-39.76** | 0.0003 |
| 320 | NM_172437 | Pus7l | Pseudouridylate synthase 7 homolog -like | **-37.04** | p<0.0001 | **-27.70** | 0.0001 |
| 321 | NM_029420 | Giyd2 | SLX1 structure-specific endonuclease subunit homolog B | **-59.52** | 0.0001 | **-73.53** | 0.0001 |
| 322 | NM_030717 | Lactb | Lactamase, beta | **-69.93** | p<0.0001 | **-74.07** | 0.0003 |
| 323 | NM_029344 | Acyp2 | Acylphosphatase 2, muscle type | **-78.13** | p<0.0001 | **-76.34** | 0.0006 |
| 324 | NM_015754 | Rbbp9 | Retinoblastoma binding protein 9 | **-88.50** | 0.0001 | **-67.11** | 0.0003 |
| 325 | NM_020000 | Med8 | Mediator of RNA polymerase II transcription, subunit 8 homolog | **-188.68** | p<0.0001 | **-136.99** | 0.0016 |
